# Tissue Distribution of 5-Hydroxymethylcytosine and Search for Active Demethylation Intermediates

**DOI:** 10.1371/journal.pone.0015367

**Published:** 2010-12-23

**Authors:** Daniel Globisch, Martin Münzel, Markus Müller, Stylianos Michalakis, Mirko Wagner, Susanne Koch, Tobias Brückl, Martin Biel, Thomas Carell

**Affiliations:** 1 Center for Integrated Protein Science (CiPSM) at the Department of Chemistry, Ludwig-Maximilians-University, Munich, Germany; 2 Center for Integrated Protein Science (CiPSM) at the Department of Pharmacy, Ludwig-Maximilians-University, Munich, Germany; University of Wales Bangor, United Kingdom

## Abstract

5–Hydroxymethylcytosine (hmC) was recently detected as the sixth base in mammalian tissue at so far controversial levels. The function of the modified base is currently unknown, but it is certain that the base is generated from 5-methylcytosine (mC). This fuels the hypothesis that it represents an intermediate of an active demethylation process, which could involve further oxidation of the hydroxymethyl group to a formyl or carboxyl group followed by either deformylation or decarboxylation. Here, we use an ultra-sensitive and accurate isotope based LC-MS method to precisely determine the levels of hmC in various mouse tissues and we searched for 5–formylcytosine (fC), 5-carboxylcytosine (caC), and 5–hydroxymethyluracil (hmU) as putative active demethylation intermediates. Our data suggest that an active oxidative mC demethylation pathway is unlikely to occur. Additionally, we show using HPLC-MS analysis and immunohistochemistry that hmC is present in all tissues and cell types with highest concentrations in neuronal cells of the CNS.

## Introduction

In 2009 it was discovered that the genetic material contains aside from the nucleobases A, C, G, T, and 5-methylcytosine (mC) the additional sixth base 5-hydroxymethylcytosine (hmC) [1], [2]. Whereas it is well established that mC is a crucial epigenetic marker [3], the function of the sixth base hmC is currently unknown, but it is speculated that it is involved in epigenetic regulation events or in active demethylation processes [4], [5], [6], [7], [8]. It was discovered that the base is generated from mC as precursor via oxidation (hydroxylation) by members of the TET enzyme family [2], [9]. These TET enzymes are Fe(II)-dependent α-oxoglutarate dioxygenases, which utilize molecular oxygen to hydroxylate alkyl groups to hydroxymethyl functionalities *via* a radical based mechanism [10]. It is currently hypothesized that hmC could be a base involved in epigenetic modulation of gene activity. The fact that hmC was also discovered in embryonic stem cells (ES cells) and seems to play an important role in ES cells self-renewal supports this hypothesis [9].

The base was originally detected in several tissues with the highest levels in mammalian brain. In brain tissues, between 0.4% and 0.7% of all dC bases were found to be converted into hmC [1]. These values are in good agreement with a recent, more detailed LC-MS study developed in our laboratory [11]. The amount of hmC in other tissues was initially found to be small with levels up to 0.1% but more precise quantification could not be obtained due to the sensitivity limitations associated with the early detection method. In an alternative attempt to achieve better information about the hmC content, an enzymatic approach was taken in which UDP-[^3^H]glucose was used as a marker, which was transferred onto hmC with the help of a specific glucosyltransferase. This assay provided hmC values far higher than those reported earlier [12].

**Figure 1:**
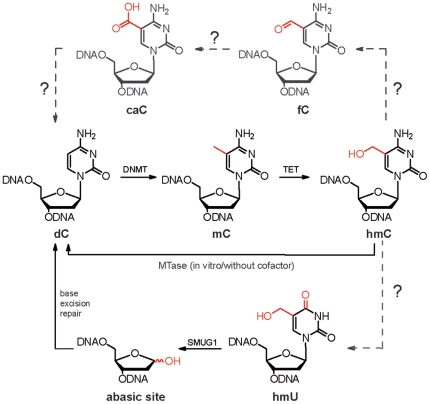
Putative demethylation pathways. Depiction of the known cytosine modifications mC and hmC and of the putative oxidative “demethylation” intermediates fC and caC. The base excision repair (BER) pathway is a second possible demethylation pathway *via* the intermediate hmU.

hmC could in principle directly modulate the binding of proteins to DNA also during epigenetic regulation processes. For example it was shown that methyl-CpG binding protein 2 (MeCP2) no longer binds to the corresponding sequences when mCs were converted to hmCs in CpG sequences [13]. Alternatively, it could function as an intermediate in active oxidative demethylation (**Figure 1**) [14]. Oxidation of the hydroxymethyl group to a formyl group would yield 5-formylcytosine (fC) which could expel formic acid and react to C. Another possibility would be further oxidation of hmC to 5-carboxylcytosine (caC) which possesses a carboxyl group and would enable quick decarboxylation to regenerate dC (**Figure 1**). Nature's most proficient enzyme orotate decarboxylase catalyzes a similar reaction in which orotate is decarboxylated to uridine [15]. Similar oxidation and decarboxylation reactions are known for thymine in the pyrimidine salvage pathway of certain eukaryotes [16], [17], [18]. Alternatively, hmC or its deamination product hmU could be substrates of specialized DNA glycosylases [6], [19], [20], [21]. Recent observations that epigenetic reprogramming is associated with the activation of base excision repair (BER) pathways support this hypothesis [22], [23]. During preparation of this manuscript a review was published, which independently postulated these two putative pathways for active DNA demethylation [24].

## Results

In order to test the idea that hmC is an intermediate of such an oxidative demethylation pathway and to shine light on the distribution of hmC in mammalian tissues we applied an isotope based HPLC-MS method to quantify hmC and to detect the presence of fC, caC, and hmU in various tissues. Mass spectrometry is a highly sensitive detection method that is, however, not quantitative. (In certain cases mass spectrometry was used for quantification of nucleosides [25]). Using stable isotope-labeled reference compounds, which have the same chromatographic and ionization properties but different molecular weights, turns MS into a quantitative method [11], [26], [27], [28]. By comparing the integrals of the individual mass signals of the natural compound (amount to be determined) and labeled compound (known amount) and subsequent application of calibration curves (**Figure S1**) very precise quantification of the natural compound is achieved.

### Synthesis of modified cytosine derivatives

**Figure 2:**
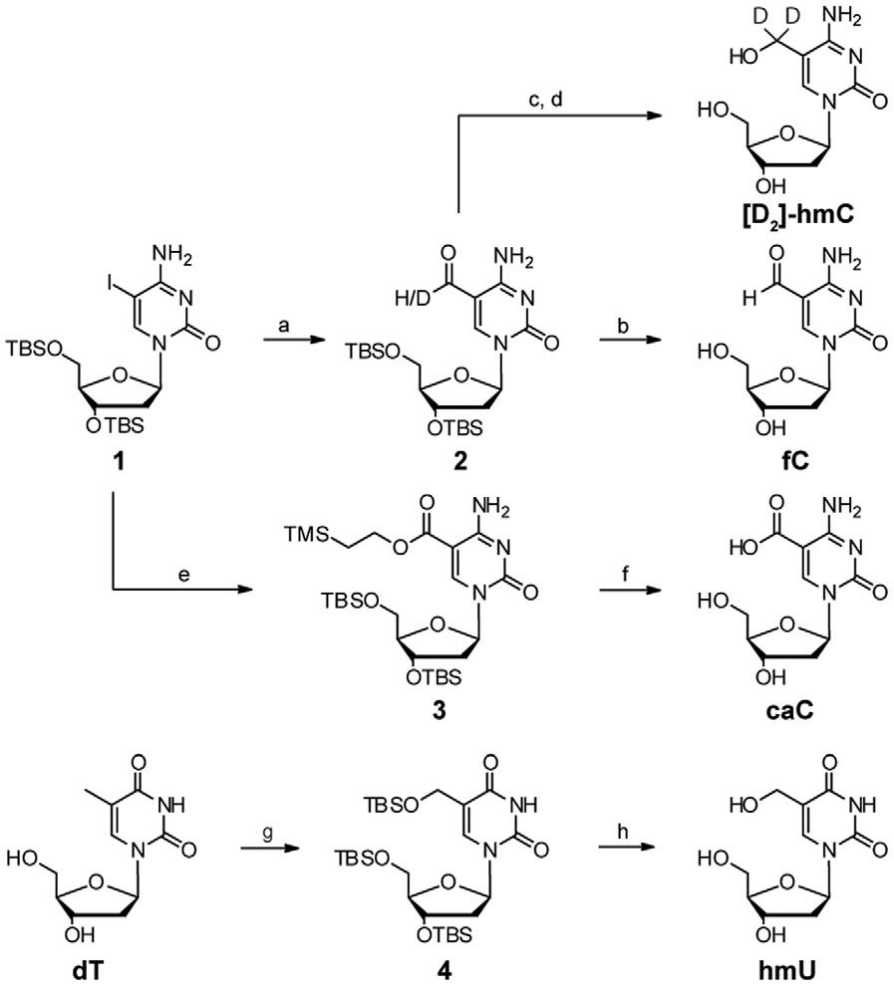
Synthesis of [D_2_]-hmC and the putative intermediates fC, caC, and hmU. a) CO, PPh_3_, Pd_2_(dba)_3_·CHCl_3_, Bu_3_SnH, 97%, with Bu_3_SnD 49%, b) HF pyridine, 75%, c) NaBD_4_, CeCl_3_·7H_2_O, 21%, d) TBAF, 51%, e) CO, TMS-Et-OH, DIPEA, Pd_2_Cl_2_(MeCN)_2_, 52%, f) TBAF, 69%, g) Reference [11] h) HF·pyridine, 70%. All reactions could also be carried out in a protective group free manner but resulted in reduced yields and tedious workups (see **Figure S2** for details).

The syntheses of the putative intermediates fC, caC, and hmU are depicted in **Figure 2**. Starting point was dC, which was first iodinated at position 5 and subsequently TBS protected to yield **1** [29]. A Pd-catalyzed carbonylation with Bu_3_SnH gave formylated compound **2**. This reaction proceeded in excellent yield even in the presence of the unprotected amine at position 4. Deprotection of **2** with HF in pyridine generated fC. hmC was easily obtained after reduction of **2** and subsequent deprotection of the TBS groups. Here, addition of a Lewis acid [30] was essential, because simple reduction using NaBH_4_ resulted mainly in decomposition of the starting material, presumably because the hydride added to the position 6 of intermediate **2**. For the synthesis of the isotope-labeled standard [D_2_]-hmC for mass spectrometric analyses Bu_3_SnD and NaBD_4_ were used in the formylation and reduction reactions, respectively (for details refer to the **Text S1** and for a protective group free synthesis see **Figure S2**). For the synthesis of caC we converted iodine **1** to a TMS-ethanol-ester in a Pd-catalyzed reaction to yield compound **3** and subsequently cleaved the protective groups. Fully protected **4** was synthesized according to a literature procedure and converted to hmU with HF·pyridine [11].

### HPLC-MS Quantification of hmC and mC in Mammalian Tissue

**Figure 3:**
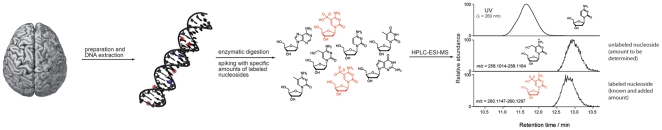
Workflow of the HPLC-MS quantification method. DNA is extracted from any kind of tissue and subsequently enzymatically digested to the nucleosides. Subsequently, a known amount of the stable isotope-labeled standard nucleoside is added. In the HPLC-MS analysis one signal for the natural (light) and one for the synthetic (heavy) compound is detected in each experiment. Quantification is performed by comparing the integrals of the specific high resolution ion current of the natural compound (amount to be determined) with their corresponding heavy atom labeled derivative (known amount).

**Figure 4:**
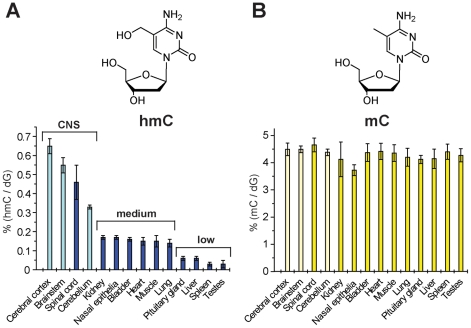
Quantification of hmC and mC in mouse tissue. A) Values of hmC in % of dG. B) Values of mC in % of dG. A)+B) Data represent values for each tissue of at least two mice with standard deviation (SD) (See **Table S1** for details). Light-colored bars represent data from our earlier study [11].

Using the LC-MS method, we quantified the levels of hmC and mC in parallel using the isotope-labeled derivative [D_2_]-hmC. This reference compound is labeled to a very high extent (>99%), which further enhances quantification accuracy of small amounts of hmC allowing us to determine the content of hmC in any tissue very precisely. For the study we isolated DNA from a variety of different mouse tissues [11]. The isotope-labeled compound [D_2_]-hmC and the obtained mixture was analyzed by HPLC-MS using a sensitive MS-detector (*Thermo Scientific LTQ Orbitrap XL*). The workflow of our method is illustrated in **Figure 3**. The distribution of hmC and mC in different mouse tissues is depicted in **Figure 4A and 4B**. We measured uniform amounts of mC that represent 4.30±0.22% of dG in all tissues in agreement with previous reports [11], [30], [31]. The only exception is nasal epithelia with a slightly lower value. To our surprise we found that hmC is present in all investigated tissues at significant quantities. More important is the discovery that the hmC values are deviating strongly in contrast to the stable amounts of mC. Three different classes of tissue are clearly distinguishable. The highest levels (0.3%–0.7%) of hmC are detected in DNA isolated from the central nervous system (CNS). The spinal cord, which is not a direct part of the brain, also belongs to hmC rich tissues with a value of 0.47%. These data strengthen the observation that neuronal tissues contain the highest levels of hmC. DNA extracted from kidney, nasal epithelium, bladder, heart, skeletal muscle, and lung has medium hmC values from 0.15%–0.17%. DNA from liver, spleen, and the endocrine glands (testes and pituitary gland) possess the lowest amounts of hmC with levels ranging from 0.03%–0.06%. This last class of tissues contains up to five times less hmC compared to tissues with medium levels and up to 20 times less than cerebral cortex as part of CNS. Interestingly, the pituitary gland, which is located in the brain, has a low hmC value of only 0.06%, supporting the hypothesis that high hmC content is related to neuronal function, rather than mere localization in the brain. In contrast to mC, the hmC amounts are tissue specific.

### Immunohistology experiments

**Figure 5:**
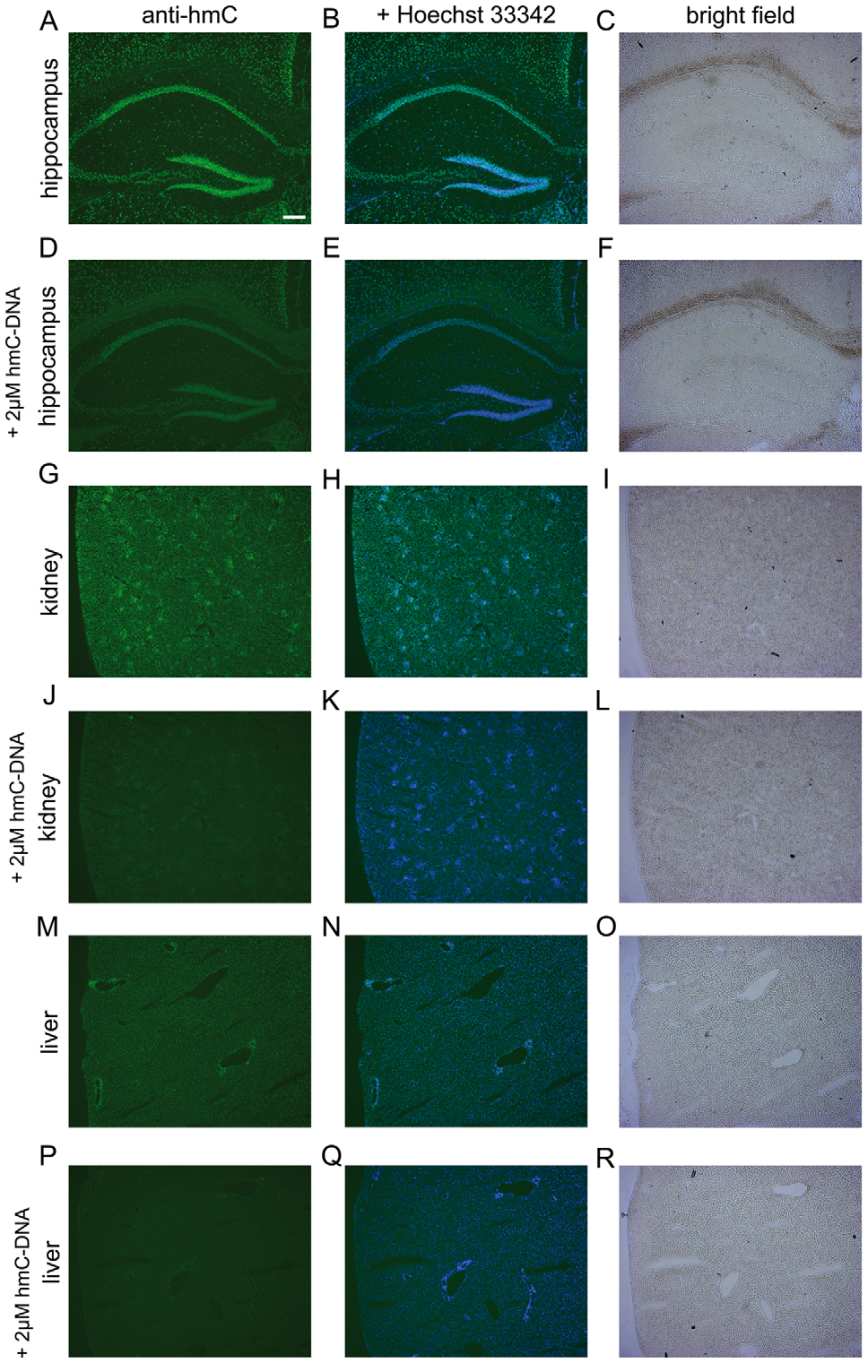
Immunolocalization of hmC in mouse hippocampus, kidney, and liver. Scale bar: 200 µM. Left column: mouse tissues stained with anti-hmC (green). Middle column: mouse tissues stained with anti-hmC (green) and Hoechst 33342 (blue) for nuclear staining. Right column: Bright field pictures of corresponding tissue. In every second row 2 µM hmC-DNA were added to compete the anti-hmC staining signal out.

**Figure 6:**
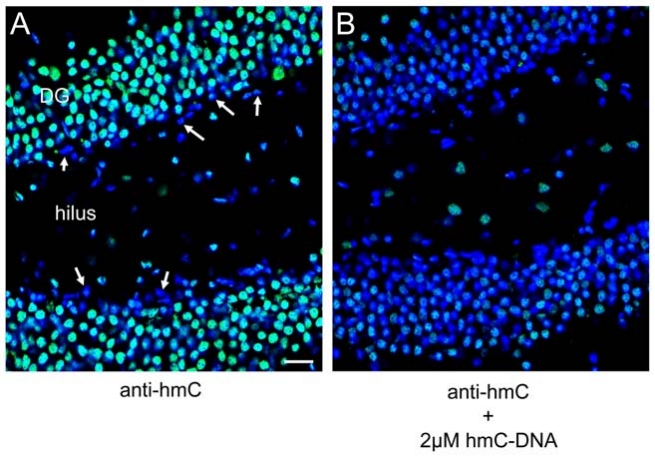
Immunolocalization of hmC in mouse hippocampus. High magnification images of hmC immunoreactivity in the dentate gyrus (DG) and the hilus of mouse hippocampus. A) Signal for anti-hmC (green) and Hoechst 33342 nuclear dye (blue) B) Competition of anti-hmC with 2 µM hmC-DNA. The scale bar marks 20 µm.

In order to further validate our quantification data and to determine the exact location of hmC in tissues more precisely we performed immunostaining experiments with a commercially available hmC-specific antibody [9], [11]. The results are depicted in **Figure 5**. The pictures show, that the sixth base is clearly located in the cell nuclei as expected. Upon addition of a hmC containing capturing oligonucleotide the signal obtained from the nuclei is reduced showing that the antibody is specific for hmC. It is evident that the highest amounts of hmC are present in the nuclei of the hippocampus and that kidney is stained with a clearly higher intensity than liver, which supports our HPLC-MS results (See **Figure S3** for further immunohistology experiments). Furthermore, virtually all cells contain hmC. Interestingly, whereas hmC is equally distributed in liver and kidney, its location in the hippocampus is very diverse. The highest levels are detected in the fully differentiated neurons of the dentate gyrus. Cells located in the subgranular zone between dentate gyrus and hilus show clearly reduced staining in line with reduced hmC levels (**Figure 6**). This area contains the neural progenitors known to generate new neurons in the hippocampus of adult mice [32], [33].

### Putative demethylation pathways

**Figure 7:**
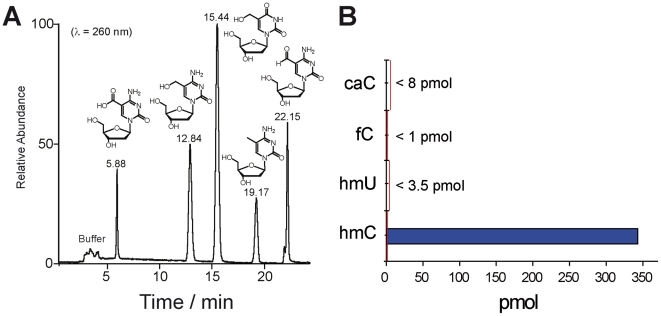
Detection of potential demethylation intermediates caC, hmU, and fC. A) HPLC-chromatogram of the synthesized cytosine and uracil modifications caC, hmC, hmU, mC, and fC as 2′-deoxynucleosides showing excellent separation of the compounds. B) Detected values of the potential intermediates as example in olfactory bulb. The red line indicates the detection limits of the modified nucleosides in enzymatically digested DNA samples.

To seek evidence if hmC is part of an oxidative demethylation pathway we investigated if the DNA samples contain fC and caC as putative downstream intermediates. Using the LC-MS method we first determined the detection limit to be in the low picomolar range, even with the background of canonical nucleosides in digested samples (**Figure 7B**). This shows that even traces of these compounds at levels 70–350 times lower than the base hmC itself could unambiguously be detected. The HPLC-chromatogram of caC, hmC, hmU, mC, and fC is shown in **Figure 7A**. In addition we maximized the amount of digested DNA applicable for HPLC-MS analysis. For these experiments, we typically used 70–160 µg of DNA isolated from the tissues. The DNA was again enzymatically digested and analyzed. For comparison, the previous experiments were performed using ten times less DNA. Despite this, neither fC nor caC could be detected. The results show that if present, fC does not reach levels above 7·10^−4^% of all nucleosides and 0.3% of hmC and caC does not reach levels above 3.5·10^−3^% of all nucleosides and 1.4% of hmC. Our results show that either an oxidative active demethylation pathway does not exist, that intermediates are short lived or that they are not released from the enzymatic complex.

Another potential active demethylation pathway could involve the base excision repair (BER) pathway of cells (**Figure 1**) either directly or via hmU [21]. Thus we investigated the presence of hmU, which could be formed by deamination of hmC by an analog of activation induced cytidine deaminase (AID) [6]. hmU is known to be a substrate for the glycosylase SMUG1 [19], [20]. It can be speculated that deamination of hmC to give hmU could be a signal for repair driven removal of mC via hmC. Again our HPLC-MS method did not provide any evidence for the presence of this compound in the digested DNA making such a mechanism equally unlikely (**Figure 7B**).

## Discussion

The role of mC in epigenetic regulatory processes is today quite well understood. The introduction and heredity of methylation patterns by DNA methyltransferases is an established fact. However, the removal of these epigenetic marks from the genetic code is still controversially discussed [24]. Thus it is not surprising that the discovery of hmC and the modifying TET enzymes in 2009 caused significant excitement. To elucidate the function of hmC in mammalian tissue, the knowledge of its distribution could provide valuable information.

### How is hmC distributed in mammalian tissues?

Our quantification results in different tissues show that hmC is present in every tissue investigated. However, our obtained values are in disagreement with the recently determined higher levels measured using an enzymatic method [12] but they are in good agreement with those levels reported initially based on radioactive labeled gel chromatography even though the estimated levels were at that time close to the detection limit of the method [1]. By employing our HPLC-MS method, that uses an internal standard and calibration curves, we solve this scientific controversy and show that hmC is present in cells in levels between 0.03% and 0.69% of dG.

Our immunohistology data support the idea that oxidation of mC to hmC is a process occurring in all cells. Interestingly, we found indication in the dentate gyrus of the hippocampus that the hmC levels are significantly lower in stem cell rich areas.

### Is hmC an intermediate in active cytosine demethylation?

It was speculated that hmC could represent an intermediate of an oxidative mC removal pathway. We proposed that hmC can be further oxidized to fC and caC similar to the pyrimidine salvage pathway or that it could be deaminated to hmU to be further excised by BER enzymes. Based on our data, however, we can exclude substantial oxidation of hmC to fC or caC or deamination of hmC to result in hmU. This does not disprove that these pathways do exist since we cannot exclude that the application of more sensitive methods, such as HPLC-MS/MS may lead to the discovery of these modifications in the future. In addition, if hmC or hmU were excised directly from DNA, it should be possible to detect these degradation products in urine [34]. But the data show that these unavoidable intermediates do not accumulate to any significant level, making it unlikely that such reactions occur at large scale or that they are extremely short lived intermediates.

## Materials and Methods

### General methods

All non-aqueous reactions were performed using flame- or ovendried glassware under an atmosphere of dry nitrogen. Commercial reagents from Sigma-Aldrich or Acros were used as received unless otherwise noted. Non-aqueous reagents were transferred under nitrogen with a syringe or cannula. Solutions were concentrated *in vacuo* on a *Heidolph* rotary evaporator. Chromatographic purification of products was accomplished using flash column chromatography on *Merck* Geduran Si 60 (40–63 µM) silica gel (normal phase), *Fluka* silica gel 100 C_18_-Reversed phase (15–35 µm), or preparative HPLC on a *Merck-Hitachi* system (L-7150 pump, L-7420 detector) equipped with a *Macherey Nagel* VP 250/32 Nucleosil 100-7 C18 column, 15.0 mL/min flow rate, as indicated. Thin layer chromatography (TLC) was performed on *Merck* 60 (silica gel F254) plates. Visualization of the developed chromatogram was performed using fluorescence quenching or anisaldehyde staining. ^1^H and ^13^C NMR spectra were recorded in deuterated solvents on *Bruker ARX 300*, *Varian VXR400S*, *Varian Inova 400* and *Bruker AMX 600* spectrometers and calibrated to the residual solvent peak. Multiplicities are abbreviated as follows: s  =  singlet, d  =  doublet, t  =  triplet, q  =  quartet, m  =  multiplet. ^13^C-NMR signals of carbons carrying deuterium atoms have been determined by C-H correlation spectra (HMBC). ESI spectra and high-resolution ESI spectra were obtained on the mass spectrometer *Thermo Finnigan* LTQ FT-ICR. Acetonitrile (HPLC gradient grade) for HPLC-ESI-MS analysis was purchased from VWR. HCOOH (p.a. for mass spectrometry) was purchased from Fluka. Mouse tissues from three male mice (C57BL/6N) were frozen in liquid nitrogen right after sacrifice. Depending on the tissue type each mouse supplied enough DNA for up to 4 measurements.

### DNA extraction from mouse tissue

DNA isolation was performed on the basis of the QIAamp DNA Mini Kit. Instead of column purification phenol extraction was performed as outlined in the next paragraph. The RNA digest was executed twice. All other steps were performed as described by the manufacturer. For samples with more than 25 mg weight the quantities of the reagents were increased accordingly. Tissue samples were homogenized with PBS and a stainless steel bead in a TissueLyser (Qiagen, 30 Hz, 2 min). ATL and proteinase K were added and the solution was incubated. DNase-free RNase A (4 µL, 100 mg/mL) was added. After mixing the sample was incubated and shaken (600 rpm) at rt for 5 min. A second portion of DNase-free RNase A (4 µL, 100 mg/mL) was added and the mixture was again incubated and shaken (600 rpm) at rt for 5 min. The tube was centrifuged briefly and buffer AL was added. The sample was mixed and incubated. Following this step the sample was no longer processed on the basis of the QIAamp DNA Mini Kit.

The sample was distributed equally to two 2 mL reaction tubes, if necessary. A 1/1 mixture of Roti®Phenol/chloroform (1 Vol.) was added and the tube was shaken vigorously at rt for 5 min. The tube was centrifuged (12100 g, 15 min) and the aq. layer was collected. This procedure was repeated once. To the obtained aq. layer chloroform (1 Vol.) was added and the tube was shaken at rt for 1 min. After centrifugation (12100 g, 5 min) the aq. layer was collected. During collection of the aq. layers special care was taken to include the interphase. The sample was distributed equally to two 2 mL reaction tubes, if necessary. Ethanol (3 Vol.) was added. The sample was left to stand at rt for approximately 2 h. After precipitation of the DNA the tube was centrifuged (12100 g, 30 min). The supernatant was discarded and the pellet was dried. Subsequently, it was dissolved in water (100–400 µL). The solution was centrifuged (12100 g, 30 min) and the supernatant was collected.

### Enzymatic digestion

For the enzymatic digestion DNA mixtures (4 to 10 µg in a final volume of 100 µL H_2_O) were heated to 100°C for 5 min to denature the DNA and rapidly cooled on ice. Buffer A (10 µL, 300 mM ammonium acetate, 100 mM CaCl_2_, 1 mM ZnSO_4_, pH 5.7) and nuclease S1 (80 units, *Aspergillus oryzae*) were added to the mixture and incubated for 3 h at 37°C. Addition of buffer B (12 µL, 500 mM Tris-HCl, 1 mM EDTA), antarctic phosphatase (10 units), snake venom phosphodiesterase I (0.2 units, *Crotalus adamanteus venom*) and incubation for further 3 h at 37°C completed the digestion. Labeled nucleosides [D_2_]hmC and [D_3_]mC were added, followed by centrifugation of the sample (12100 g, 15 min). The supernatant was removed, the volume reduced to 100 µL and measured with HPLC-ESI-MS. Each sample was analyzed at least in duplicate with independent concentrations of the two labeled nucleosides. The concentrations of standard solutions were chosen to be in the expected range of the sample nucleoside concentration.

### HPLC-ESI-MS

The samples (100 µL injection volume) were analyzed by HPLC-ESI-MS on a *Thermo Finnigan LTQ Orbitrap XL* and chromatographed by a *Dionex Ultimate 3000 HPLC* system with a flow of 0.15 mL/min over an Uptisphere120-3HDO column from *Interchim*. The column temperature was maintained at 30°C. Eluting buffers were buffer C (2 mM HCOONH_4_ in H_2_O (pH 5.5)) and buffer D (2 mM HCOONH_4_ in H_2_O/MeCN 20/80 (pH 5.5)). The gradient was 0 → 12 min; 0% → 3% buffer D; 12 → 60 min; 3% → 60% buffer D; 60 → 62 min; 60% → 100% buffer D; 62 → 70 min; 100% buffer D; 70 → 85 min; 100 → 0% buffer D; 85 → 95 min; 0% buffer D. The elution was monitored at 260 nm (*Dionex Ultimate 3000 Diode Array Detector*). The chromatographic eluent was directly injected into the ion source without prior splitting. Ions were scanned by use of a positive polarity mode over a full-scan range of *m/z* 200–1000 with a resolution of 30.000. Parameters of the mass spectrometer were tuned with a freshly mixed solution of adenosine (5 µM) in buffer C. The parameters used in this section were sheath gas flow rate, 16 arb; auxiliary gas flow rate, 11 arb; sweep gas flow rate, 4 arb; spray voltage, 5.0 kV; capillary temperature, 200°C; capillary voltage, 12 V, tube lens 60 V.

### Mass calibration curves

Mass calibration curves of the labeled and corresponding unlabeled synthesized nucleosides were obtained at five different concentration ratios. For each concentration an average value of three independent measurements was determined (**Figure S1**). Each labeled nucleoside solution was mixed with three solutions with different concentrations of the corresponding unlabeled nucleosides. The areas of labeled and unlabeled nucleosides of the LC-MS measurements were determined using the *Qualbrowser* program by extraction of the accurate mass with a mass filter (**Table S1**) from the total ion current (TIC). The linear fits of the determined area ratios over the amount ratios gave R^2^-values of minimum 0.9987 for mC and 0.9997 for hmC. The linear equations were used for calculation of the exact nucleoside contents in genomic DNA samples. Synthetic labeled nucleosides were added to the enzymatically digested nucleoside mixture and the areas of labeled and unlabeled nucleosides were determined as described above. The amount of each nucleoside was calculated from the obtained area ratios and the linear fit equations of the calibration curves. Determination of the precise amounts of mC and hmC by the described isotope-labeled mass spectrometric method and determination of the precise amount of dG by integration of the UV/vis signal of the HPLC chromatogram enabled calculation of the exact percentage of mC and hmC to dG [11]. We have chosen dG as a standard, because it forms base pairs with all three cytosine derivatives dC, mC and hmC.

### Immunohistochemistry

Coronal cryosections (12 µm) from 12 week old C57-BL6/N mice were rehydrated in phosphate buffered saline (PBS), fixed (10 min, 4% paraformaldehyde in PBS, pH 7,4), treated with 2N HCl in PBS (20 min) and incubated for 16 hours (4°C) with primary antibodies in 5% chemiblocker (Millipore, Germany) and 0.3% Triton X-100 in PBS. The primary antibody used was: rabbit anti-5-hydroxymethylcytosine (hmC, 1∶500, Active Motif, Belgium). For secondary detection we used goat Alexa488 anti-rabbit (1∶800, Cell Signaling Technologies, Germany). Cell nuclei were counterstained with Hoechst 33342 (5 µg/mL, Sigma, Germany) and sections were mounted with aqueous mounting medium (PermaFluor, Beckman-Coulter, USA). Tissues were analyzed using a Zeiss Axioscope epifluorescence microscope equipped with a HBO 100 mercury arc lamp, appropriate filters equipped with an MRc ccd camera (Zeiss, Germany). Laser scanning confocal micrographs were collected using a LSM 510 meta microscope (Carl Zeiss, Germany).

## Supporting Information

Figure S1 **Mass calibration curves of the nucleosides mC and hmC.** Linear fits of five data points represent perfect linearity with R^2^-values of 0.9987 for mC and 0.9997 for hmC.
(TIF)

Figure S2 **Protective group free synthesis of putative cytosine derivatives.** a) I_2_, *m*CPBA, 63% b) CO, Bu_3_SnH, Pd_2_(dba)_3_, 35% c) NaBH_4_, CeCl_3_·7H_2_O, 53% d) TMS-Et-OH, CO, DIPEA, PdCl_2_(MeCN)_2_, 22% e) HF·pyr, 11%. The putative oxidative catabolites of hmC could also be synthesized in a protective group free manner. However, these syntheses suffer from lower yields and the workup procedures get considerably more tedious than with the TBS-protected compounds. dC can be converted to **5** by oxidative iodination using iodine and *m*CPBA. Subsequent palladium catalyzed formylation with CO and Bu_3_SnH in DMF as solvent gave fC in 34% yield. Subsequent Luche reduction furnished hmC. **5** could be converted to **6** in a carbonylating coupling of the iodine with TMS-ethanol. Final cleavage of the ester with F^-^ yields ethene, TMSF and caC.
(TIF)

Figure S3 **Immunolocalization of hmC in mouse kidney, liver, and heart.** Scale bar: 20 µm. Mouse tissues stained with anti-hmC (green), Hoechst 33342 nuclear staining is shown in blue. A+G+M) anti-hmC staining. B+H+N) anti-hmC (green) and Hoechst 33342 (blue). D+J+P) The anti-hmC staining signal was competed out by 2µM hmC-DNA. E+K+Q) The anti-hmC staining signal was competed out by 2µM hmC-DNA. C+F+I+L+O+R) Bright field pictures of the used tissues. gl: glomerolus, s: sinusoid.
(TIF)

Table S1 **Nucleoside percentages of mC and hmC to dG in different mouse tissues** (***Figure 2*** **in main text).** Each mouse tissue is listed with the determined values, the standard deviation (SD) and the relative standard deviation (RSD). The average mouse values are listed in the last column. For muscle, the DNA of mouse 1+2 was mixed due to a low amount of DNA. The applied mass ranges of analyzed nucleosides are shown as well.
(PDF)

Text S1 **Supporting text.**
(PDF)
