# Supplementary figures and images for: Tissue Distribution of 5-Hydroxymethylcytosine and Search for Active Demethylation Intermediates
Source: PLoS One. 2010 Dec 23;5(12):e15367. doi: 10.1371/journal.pone.0015367 (PMC3009720; doi:10.1371/journal.pone.0015367)

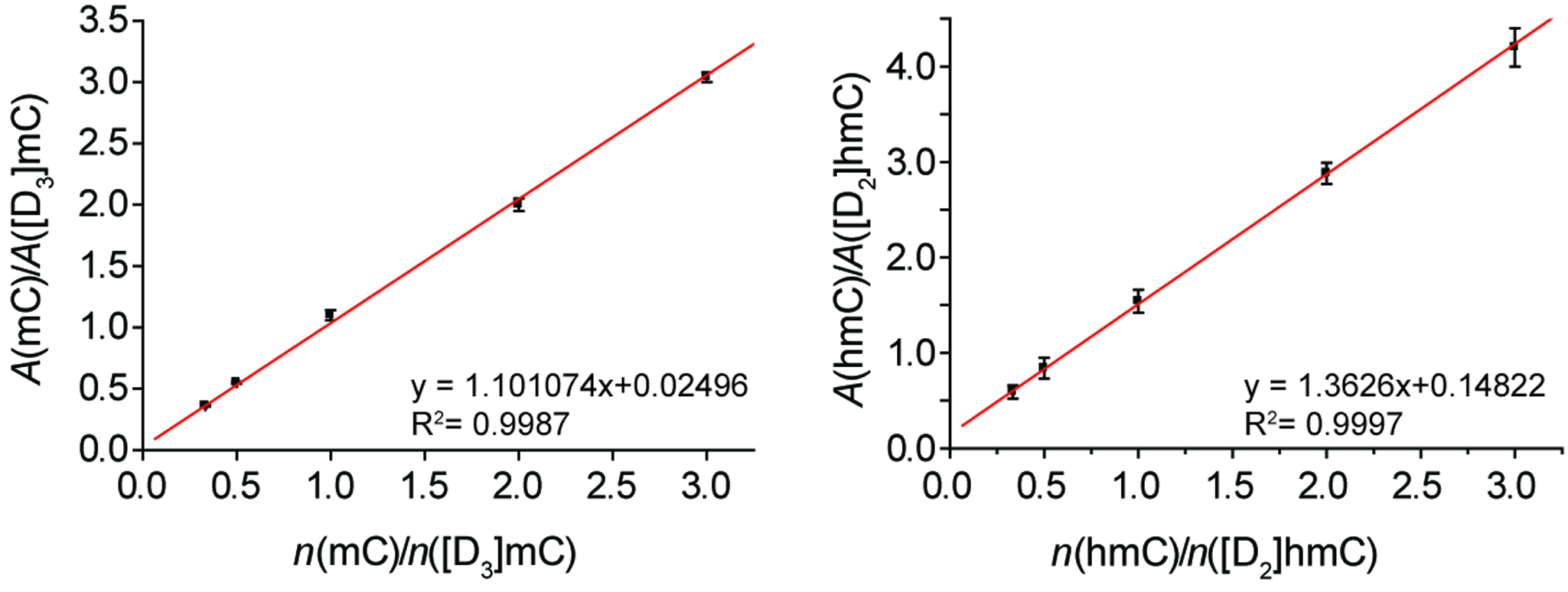

Supplement: Figure S1 — Mass calibration curves of the nucleosides mC and hmC. Linear fits of five data points represent perfect linearity with R2-values of 0.9987 for mC and 0.9997 for hmC. (TIF) [file pone.0015367.s001.tif]

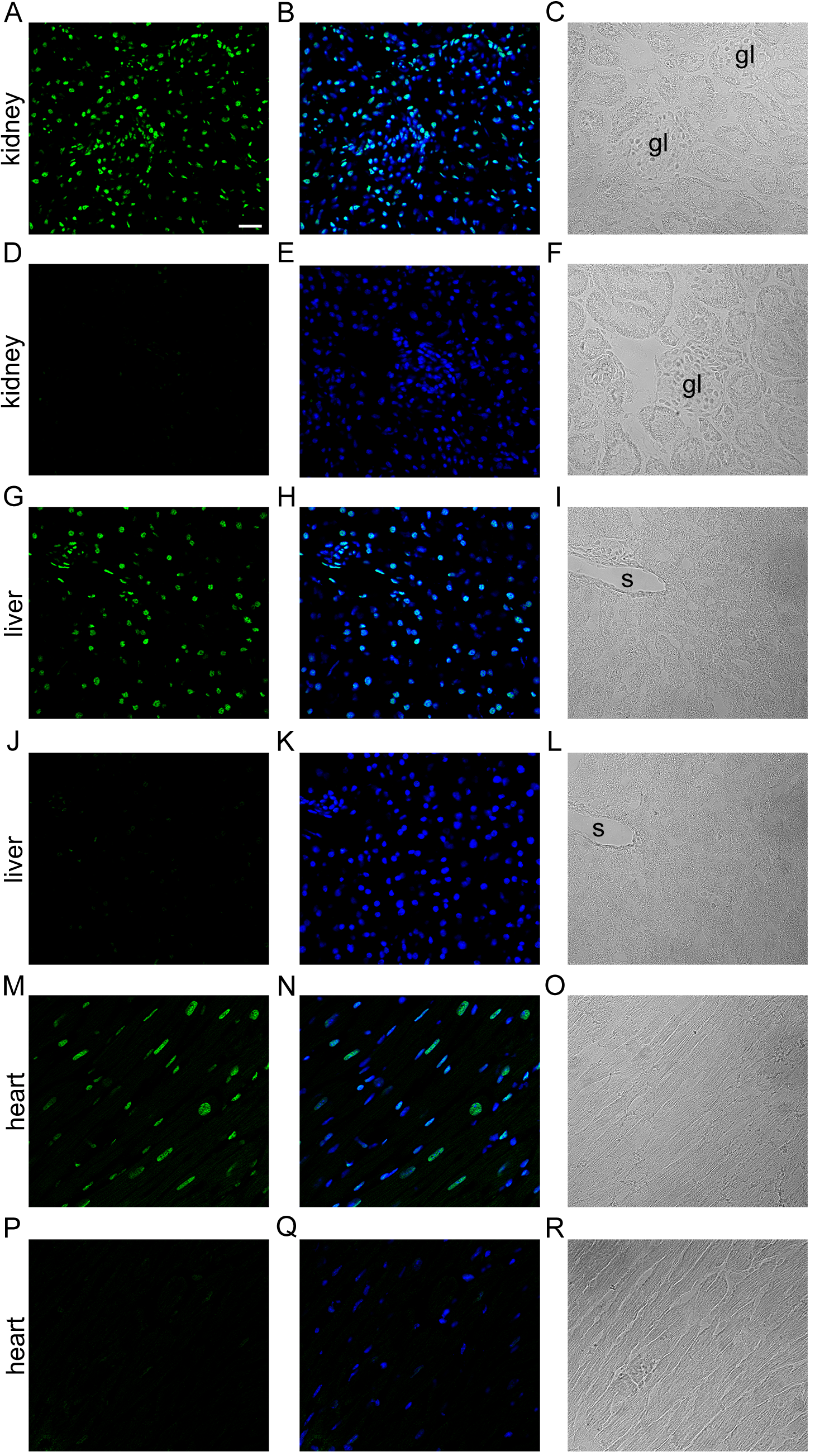

Supplement: Figure S3 — Immunolocalization of hmC in mouse kidney, liver, and heart. Scale bar: 20 µm. Mouse tissues stained with anti-hmC (green), Hoechst 33342 nuclear staining is shown in blue. A+G+M) anti-hmC staining. B+H+N) anti-hmC (green) and Hoechst 33342 (blue). D+J+P) The anti-hmC staining signal was competed out by 2µM hmC-DNA. E+K+Q) The anti-hmC staining signal was competed out by 2µM hmC-DNA. C+F+I+L+O+R) Bright field pictures of the used tissues. gl: glomerolus, s: sinusoid. (TIF) [file pone.0015367.s003.tif]
