# Supplementary material for: Tissue Distribution of 5-Hydroxymethylcytosine and Search for Active Demethylation Intermediates
Source: PLoS One. 2010 Dec 23;5(12):e15367. doi: 10.1371/journal.pone.0015367 (PMC3009720; doi:10.1371/journal.pone.0015367)
Supplement: Table S1 — Nucleoside percentages of mC and hmC to dG in different mouse tissues ( Figure 2 in main text). Each mouse tissue is listed with the determined values, the standard deviation (SD) and the relative standard deviation (RSD). The average mouse values are listed in the last column. For muscle, the DNA of mouse 1+2 was mixed due to a low amount of DNA. The applied mass ranges of analyzed nucleosides are shown as well. (PDF) [file pone.0015367.s004.pdf]

Mouse tissue (mC content)		Mouse 1	Mouse 2	Mouse 3	Average mouse 1-3
Spinal cord		4.55	4.82	-	**4.66**
	SD	0.19	0.23	-	0.25
	RSD / %	4.09	4.86	-	5.29
Kidney		4.67	3.51	4.76	**4.13**
	SD	0.12	0.17	0.15	0.64
	RSD / %	2.66	4.94	3.17	15.54
Nasal epithelia		3.80	3.66	-	**3.73**
	SD	0.11	0.24	-	0.20
	RSD / %	2.89	6.51	-	5.31
Bladder		4.26	4.24	4.66	**4.38**
	SD	0.09	0.05	0.46	0.33
	RSD / %	2.11	1.20	9.93	7.63
Heart		4.55	4.51	4.23	**4.42**
	SD	0.45	0.20	0.06	0.30
	RSD / %	9.94	4.48	1.47	6.76
Muscle		4.03		4.62	**4.36**
	SD	0.08		0.23	0.31
	RSD / %	1.98		4.94	7.19
Lung		4.57	4.13	4.15	**4.21**
	SD	0.49	0.07	0.20	0.33
	RSD / %	10.82	1.69	4.80	7.73
Pituitary gland		4.14	4.11	-	**4.13**
	SD	0.20	0.08	-	0.15
	RSD / %	4.75	0.07	-	3.63
Liver		4.37	4.78	-	**4.15**
	SD	0.02	0.21	-	0.36
	RSD / %	0.43	4.31	-	8.65
Spleen		4.65	4.25	4.31	**4.41**
	SD	0.35	0.03	0.06	0.28
	RSD / %	7.43	0.59	1.44	6.40
Testes		4.43	4.27	4.01	**4.28**
	SD	0.29	0.01	-	0.24
	RSD / %	6.54	0.14	-	5.58

**Mouse tissue (hmC content)**		**Mouse 1**	**Mouse 2**	**Mouse 3**	**Average mouse 1-3**
Spinal cord		0.40	0.57	-	**0.46**
	SD	0.05	0.01	-	0.09
	RSD / %	12.30	1.71	-	19.55
Kidney		0.19	0.16	0.18	**0.17**
	SD	0.01	<0.01	<0.01	0.01
	RSD / %	2.68	0.75	0.01	3.70
Nasal epithelia		0.16	0.18	-	**0.17**
	SD	<0.01	<0.01	-	0.01
	RSD / %	0.64	1.56	-	7.40
Bladder		0.15	0.16	0.17	**0.16**
	SD	0.01	<0.01	<0.01	0.01
	RSD / %	5.01	3.16	0.36	5.24
Heart		0.15	0.17	0.14	**0.15**
	SD	0.01	0.02	<0.01	0.02
	RSD / %	6.97	14.07	0.25	11.82
Muscle		0.16		0.13	**0.15**
	SD	0.03		0.01	0.03
	RSD / %	18.86		8.42	22.35
Lung		0.13	0.14	0.16	**0.14**
	SD	<0.01	0.02	0.01	0.02
	RSD / %	1.87	16.18	8.32	14.71
Pituitary gland		0.07	0.04	-	**0.06**
	SD	<0.01	<0.01	-	0.01
	RSD / %	7.07	6.53	-	18.71
Liver		0.06	0.06	-	**0.06**
	SD	0.01	0.01	-	0.01
	RSD / %	18.12	14.82	-	16.75
Spleen		0.03	0.04	0.02	**0.03**
	SD	0.01	0.01	-	0.01
	RSD / %	22.54	22.39	-	32.55
Testes		0.02	0.04	0.03	**0.03**
	SD	-	-	0.02	0.02
	RSD / %	-	-	81.42	58.06

Nucleoside	Nucleoside Mass range *m/z*	Labeled nucleoside Mass range *m/z*
hmC	258.1024-258.1144	260.1167-260.1277
mC	242.1075-242.1195	245.1261-245.1381
