# Supplementary material for: Tissue Distribution of 5-Hydroxymethylcytosine and Search for Active Demethylation Intermediates
Source: PLoS One. 2010 Dec 23;5(12):e15367. doi: 10.1371/journal.pone.0015367 (PMC3009720; doi:10.1371/journal.pone.0015367)
Supplement: Text S1 — Supporting text. (PDF) [file pone.0015367.s005.pdf]

Synthetic Procedures

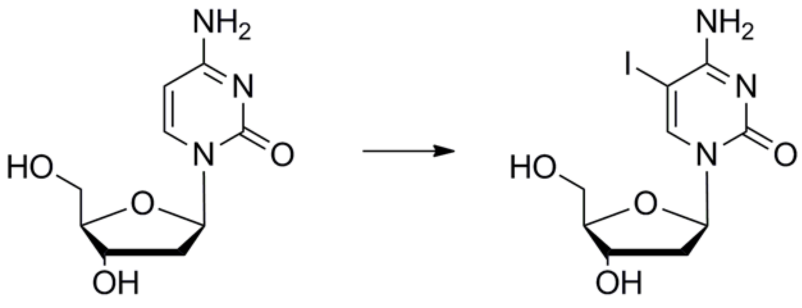

dC **5**

In a flame dried round bottom flask 10.0 g **dC** (44.0 mmol, 1.0 eq), 7.70 g iodine (26.4 mmol, 0.6 eq) and 11.4 g *m*CPBA (70 %, 46.2 mmol, 1.05 eq) were dissolved in 120 mL DMF. The reaction mixture was stirred 2 h at room temperature and subsequently evaporated to dryness. Purification by column chromatography (DCM/MeOH/H_2O/NH_3 190:10:0.6:0.6 → 90:10:0.6:0.6) yielded 9.71 g (63 %) of **5** as an orange solid.

^{1}H NMR (400 MHz, $CDCl_3$/MeOD) δ (ppm) = 8.46 (s, 1H), 6.13 (t, ^3J=6.0, 1H), 4.34 (dt, ^3J=4.7, ^3J=6.3 , 1H), 3.93 (dt, ^3J=3.0, ^3J=4.3, 1H), 3.84 (dd, ^3J=3.0 Hz, ^2J=12.1, 1H), 3.72 (dd, ^3J=3.2, ^2J=12.1, 1H), 2.39 (ddd, ^3J=4.8, ^3J=6.3, ^2J=13.7, 1H), 2.20 – 2.09 (m, 1H). ^{13}C NMR (101 MHz, MeOD) δ (ppm) = 163.9, 153.9, 150.9, 89.5, 88.3, 71.5, 62.2, 56.2, 42.5. HRMS (ESI +) calculated for $C_9H_{13}IN_3O_4^+$ $[M+H]^+$: 353.9945, found: 353.9944.

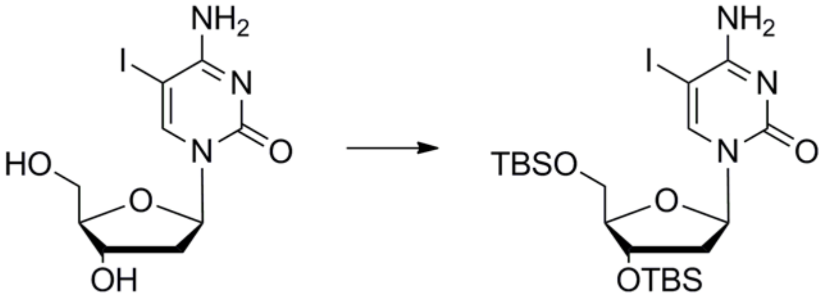

5 **1**

In a flame dried round bottom flask 5.00 g **5** (13.5 mmol, 1.0 eq), 4.16 g imidazole (60.5 mmol, 4.5 eq) and 6.24 g (40.4 mmol, 3.0 eq) TBS-Cl were dissolved in 80 mL

DMF and stirred at RT for 16 h. Subsequently the reaction was stopped by the addition of 150 mL sat. $NaHCO_3$ and extracted with 300 mL chloroform. The organic layers were washed with 300 mL H_2O, dried over $MgSO_4$ and the solvent removed *in vacuo*. The crude product was purified by column chromatography (DCM/MeOH 99:1 → 49:1) to yield 6.25 g (80 %) of **1** as a slightly yellow solid.

^{1}H NMR (400 MHz, $CDCl_3$) δ (ppm) = 8.06 (s, 1H), 6.25 – 6.19 (m, 1H), 4.34 (dt, ^3J=2.9, ^3J=5.9, 1H), 3.97 (q, ^3J=2.6, 1H), 3.87 (dd, ^3J=2.6, ^2J=11.4, 1H), 3.74 (dd, ^3J=2.6, ^2J=11.4, 1H), 2.44 (ddd, ^3J=3.0, ^3J=5.9, ^2J=13.3, 1H), 2.00 – 1.90 (m, 1H), 0.92 (s, 9H), 0.87 (s, 9H), 0.13 (s, 3H), 0.12 (s, 3H), 0.06 (s, 3H), 0.05 (s, 3H). ^{13}C NMR (101 MHz, $CDCl_3$) δ (ppm) = 163.2, 154.3, 146.7, 88.3, 86.8, 72.2, 62.8, 56.2, 42.6, 26.1, 25.7, 18.5, 18.0, -4.6, -4.9, -5.2, -5.3. HRMS (ESI+): calculated for $C_{21}H_{41}IN_3O_4Si_2^+$ [M+H]$^+$: 582.1675, found: 582.1683.

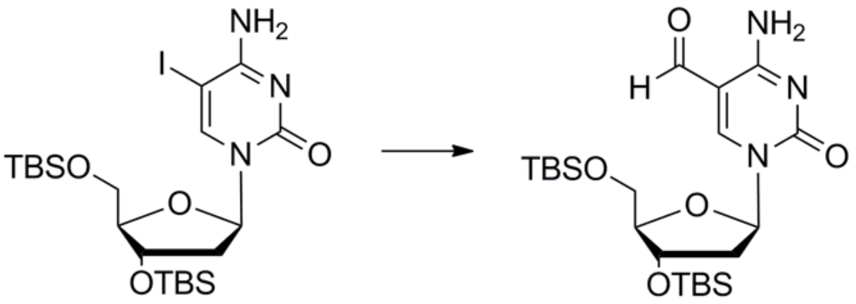

1 **2**

In a high pressure glass autoclave 3.50 g **1** (6.02 mmol, 1.0 eq), 947 mg PPh_3 (3.61 mmol, 0.6 eq) and 623 mg $Pd_2(dba)_3$*$CHCl_3$ (0.60 mmol, 0.1 eq) were dissolved in 90 mL toluene. The autoclave was flushed with CO twice to remove residual air and subsequently the reaction stirred at a CO pressure of 3.5 bar at 60 °C. With a syringe pump 2.02 mL Bu_3SnH (7.22 mmol, 1.2 eq) were added through a septum at 0.3 mL per hour. After complete addition the reaction mixture was stirred additional 12 hours at 60 °C. Subsequently the CO was discharged and the solvent evaporated *in vacuo*. The crude product was purified by column chromatography (*i*Hex/EtOAc 4:1→2:1→1:1) to yield 2.84 g (97 %) **2** as a yellow solid.

^{1}H NMR (300 MHz, $CDCl_3$) δ (ppm) = 9.51 (s, 1H), 8.57 (s, 1H), 8.37 (s, 1H), 7.46 (s, 1H), 6.19 (t, ^3J=6.1, 1H), 4.40 – 4.32 (m, 1H), 4.08 – 4.02 (m, 1H), 3.95 (dd, ^3J=2.7,

^{2}J=11.7, 1H), 3.78 (dd, ^{3}J=2.6, ^{2}J=11.6, 1H), 2.59 (ddd, ^{3}J=3.6, ^{3}J=5.8, ^{2}J=10.3, 1H), 2.20 – 2.08 (m, 1H), 0.89 (s, 9H), 0.88 (s, 9H), 0.10 (s, 3H), 0.08 (s, 6H), 0.07 (s, 3H). ^{13}C NMR (75 MHz, $CDCl_3$) δ (ppm) = 187.1, 162.1, 153.1, 152.6, 104.9, 88.8, 87.9, 71.5, 62.6, 42.8, 25.9, 25.7, 18.4, 17.9, -4.5, -4.9, -5.2, -5.4. HRMS (ESI+): calculated for $C_{22}H_{42}N_3O_5Si_2^+$ [M+H]$^+$: 484.2658, found: 484.2654.

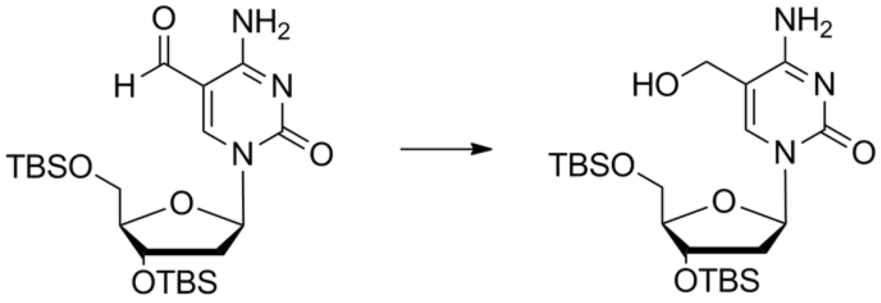

2 **7**

In a flame dried round bottom flask 300 mg **2** (0.62 mmol, 1.0 eq) and 707 mg $CeCl_3$*7 H_2O (1.86 mmol, 3.0 eq) were dissolved in 30 mL methanol. To this solution 24 mg $NaBH_4$ (0.62 mmol, 1.0 eq) were added and the mixture stirred at room temperature for 30 min. The reaction was stopped by addition of 100 mL sat. NH_4Cl and extracted with 100 mL EtOAc. Subsequently the organic layers were washed twice with 100 mL NH_4Cl, dried over $MgSO_4$, evaporated to dryness and the crude product purified by column chromatography (DCM/MeOH 19:1, dry loaded) to yield 184 mg **7** (61 %) as a colorless oil.

^{1}H NMR (599 MHz, $CDCl_3$) δ (ppm) = 7.59 (s, 1H), 6.13 (t, ^{3}J=6.4, 1H), 4.39 (d, ^{2}J=13.1, 1H), 4.36 (d, ^{2}J=13.1, 1H), 4.30 (dt, ^{3}J=3.5, ^{3}J=6.6, 1H), 3.90 (q, ^{3}J=3.1, 1H), 3.81 (dd, ^{3}J=3.2, ^{2}J=11.2, 1H), 3.72 (dd, ^{3}J=3.0, ^{2}J=11.3, 1H), 2.36 (ddd, ^{3}J=3.6, ^{3}J=6.1, ^{2}J=13.3, 1H), 1.93 (dt, ^{3}J=6.5, ^{2}J=13.2, 1H), 0.88 (s, 9H), 0.87 (s, 9H), 0.08 (s, 3H), 0.07 (s, 3H), 0.05 (s, 3H), 0.04 (s, 3H). ^{13}C NMR (151 MHz, $CDCl_3$) δ (ppm) = 165.2, 156.2, 138.6, 106.0, 87.8, 86.2, 71.7, 62.7, 59.5, 42.2, 25.9, 25.8, 18.4, 18.0, -4.6, -4.9, -5.3, -5.4. HRMS (ESI+): calculated for $C_{22}H_{44}N_3O_5Si_2^+$ [M+H]$^+$: 486.2814, found: 484.2815.

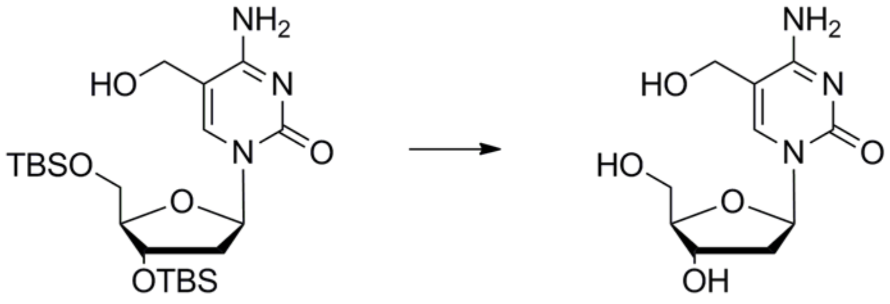

7 **hmC**

In a flame dried round bottom flask 100 mg **7** (0.21 mmol, 1.0 eq) were dissolved in 10 mL THF and 410 µL TBAF (1M in THF, 0.41 mmol, 2.0 eq) added. The solution was stirred at room temperature for 3 h. Subsequently 1 mL TMSOMe was added and the reaction mixture stirred for 30 min. The solvents were evaporated *in vacuo* and the crude product purified by HPLC (0-20 % MeCN in 45 min) to yield 27 mg (51 %) **hmC** as a colorless solid.

^{1}H NMR (400 MHz, D_2O/MeOD) δ (ppm) = 7.83 (s, 1H), 6.16 (t, ^{3}J=6.5, 1H), 4.42 – 4.25 (m, 3H), 3.95 (dd, ^{3}J=3.7, ^{3}J=8.5, 1H), 3.76 (dd, ^{3}J=3.4, ^{2}J=12.5, 1H), 3.67 (dd, ^{3}J=4.8, ^{2}J=12.5, 1H), 2.34 (ddd, ^{3}J=4.1, ^{3}J=6.4, ^{2}J=14.0, 1H), 2.24 – 2.11 (m, 1H). ^{13}C NMR (101 MHz, D_2O/MeOD) δ (ppm) = 166.1, 158.3, 141.3, 107.8, 88.0, 87.3, 71.6, 62.3, 59.0, 41.0. HRMS (ESI-): calculated for $C_{10}H_{14}N_3O_5^-$ [M-H]$^-$: 256.0939, found: 256.0936.

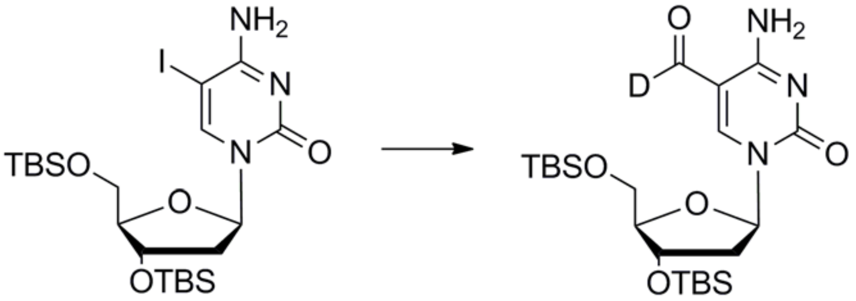

1 **8**

In a high pressure glass autoclave 200 mg **1** (0.34 mmol, 1.0 eq), 54 mg PPh_3 (0.21 mmol, 0.6 eq) and 36 mg $Pd_2(dba)_3$*$CHCl_3$ (0.03 mmol, 0.1 eq) were dissolved in 8

mL deutero-benzene. The autoclave was flushed with CO twice to remove residual air and subsequently the reaction stirred at a CO pressure of 3.5 bar at 60 °C. With a syringe pump 116 µL Bu_3SnH (0.41 mmol, 1.2 eq, diluted with 850 µL deutero-benzene) were added through a septum at 0.1 mL per hour. After complete addition the reaction mixture was stirred additional 12 hours at 60 °C. Subsequently the CO was discharged and the solvent evaporated *in vacuo*. The crude product was purified by column chromatography (*i*Hex/EtOAc 4:1→2:1→1:1) to yield 82 mg (49 %) **8** as a yellow solid.

^{1}H NMR (300 MHz, $CDCl_3$) δ (ppm) = 8.52 (s, 1H), 8.18 (s, 1H), 7.04 (s, 1H), 6.19 (t, ^3J=6.1, 1H), 4.38 – 4.31 (m, 1H), 4.03 (dd, ^3J=2.6, ^3J=5.6, 1H), 3.94 (dd, ^3J=2.6, ^2J=11.6, 1H), 3.77 (dd, ^3J=2.5, ^2J=11.6, 1H), 2.60 (ddd, ^3J=3.8, ^3J=6.1, ^2J=13.6, 1H), 2.06 (dt, ^3J=6.1, ^2J=9.6, 1H), 0.88 (2x s, 18H), 0.09 (s, 3H), 0.07 (s, 6H), 0.05 (s, 3H). ^{13}C NMR (75 MHz, $CDCl_3$) δ (ppm) = 186.5, 162.7, 153.3, 152.8, 104.9, 88.5, 87.6, 71.5, 62.5, 42.9, 25.9, 25.7, 18.4, 17.9, -4.6, -5.0, -5.3, -5.4. HRMS (ESI-): calculated for $C_{22}H_{39}DN_3O_5Si_2^-$ [M-H]$^-$: 483.2575, found: 483.2569.

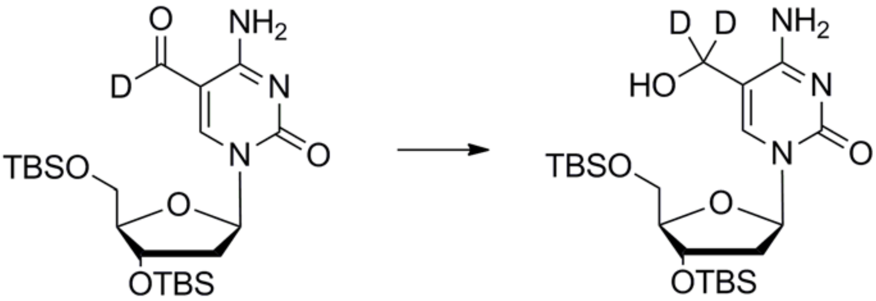

8 **9**

In a flame dried round bottom flask 75 mg **8** (0.15 mmol, 1.0 eq) and 176 mg $CeCl_3$*7 H_2O (0.46 mmol, 3.0 eq) were dissolved in 5 mL deutero-methanol. To this solution 7 mg $NaBD_4$ (0.15 mmol, 1.0 eq) were added and the mixture was stirred at room temperature for 15 min. The reaction was stopped by the addition of 50 mL sat. NH_4Cl and extracted with 50 mL EtOAc. Subsequently the organic layers were washed twice with 50 mL NH_4Cl, dried over $MgSO_4$, evaporated to dryness and the

crude product purified by column chromatography (DCM/MeOH 19:1, dry loaded) to yield 16 mg **9** (21 %) as a colorless oil.

^{1}H NMR (300 MHz, $CDCl_3$) δ (ppm) = 7.61 (s, 1H), 6.14 (t, ^3J=6.4, 1H), 4.31 (dt, ^3J=3.4, ^3J=6.4, 1H), 3.91 (dd, ^3J=3.3, ^3J=6.4, 1H), 3.83 (dd, ^3J=3.1, ^2J=11.1, 1H), 3.72 (dd, ^3J=2.9, ^2J=11.2, 1H), 2.38 (ddd, ^3J=3.5, ^3J=6.2, ^2J=13.4, 1H), 1.94 (dt, ^3J=6.6, ^2J=13.2, 1H), 0.89 (2x s, 18H), 0.08 (2x s, 6H), 0.05 (2x s, 6H). ^{13}C NMR (75 MHz, $CDCl_3$) δ (ppm) = 165.2, 156.2, 138.6, 105.8, 87.8, 86.3, 71.7, 62.7, 58.8, 42.2, 25.9, 25.8, 18.37, 18.0, -4.6, -4.9, -5.3, -5.4. HRMS (ESI+): calculated for $C_{22}H_{42}D_2N_3O_5Si_2^+$ [M+H]$^+$: 488.2940, found: 488.2941.

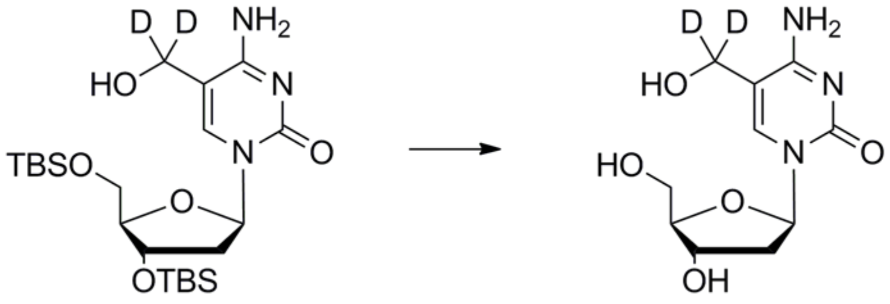

9 **[D$_2$]-hmC**

In a polypropylene tube 16 mg **9** (0.03 mmol, 1.0 eq) were dissolved in 2 mL EtOAc, subsequently 14 µL pyridine (0.17 mmol, 5.0 eq) and 14 µL HF*pyridine (70 % HF, 0.52 mml, 15.0 eq) were added and the reaction mixture stirred 14 h at room temperature. During this time a white solid precipitated. 500 µL TMSOMe were added and the reaction mixture stirred another 30 min. Subsequently the solid was collected by centrifugation (6000 rpm, 15 min). The crude product was purified by HPLC (0-25 % MeCN in 45 min) to yield 10 mg **[D$_2$]-hmC** (94 %) as a colorless solid.

^{1}H NMR (400 MHz, D_2O/MeOD) δ (ppm) = 7.92 (s, 1H), 6.28 (t, ^3J=6.6, 1H), 4.46 (dt, ^3J=3.5, ^3J=6.1, 1H), 4.07 (dd, ^3J=3.2, ^3J=6.7, 1H), 3.88 (dd, ^3J=3.3, ^2J=12.5, 1H), 3.79 (dd, ^3J=4.9, ^2J=12.8, 1H), 2.46 (ddd, ^3J=4.0, ^3J=6.4, ^2J=12.7, 1H), 2.32 (dt, ^3J=7.0, ^2J=14.2, 1H). ^{13}C NMR (101 MHz, D_2O/MeOD) δ (ppm) = 166.1, 158.4, 141.6, 107.7, 87.8, 87.3, 71.5, 62.2, 53.4, 40.5. HRMS (ESI+): calculated for $C_{10}H_{13}D_2N_3NaO_5^+$ [M+H]$^+$: 282.1029, found: 282.1030.

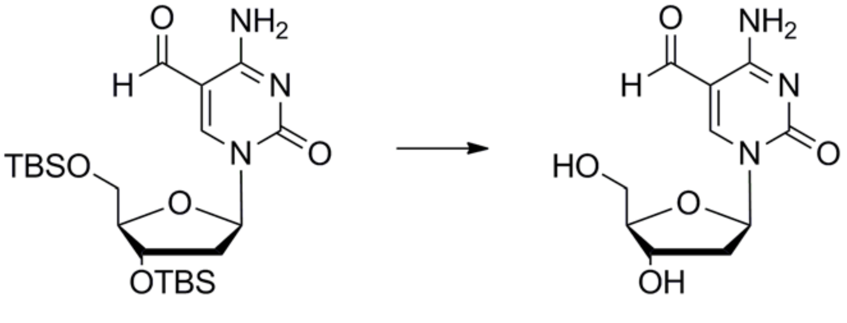

2 **fC**

In a polypropylene tube 400 mg **2** (0.78 mmol, 1.0 eq) were dissolved in 30 mL EtOAc, subsequently 316 µL pyridine (3.91 mmol, 5.0 eq) and 305 µL HF*pyridine (70 % HF, 11.7 mml, 15.0 eq) were added at 0°C and the reaction mixture stirred 14 h at room temperature. During this time a white solid precipitated. 3 mL TMSOMe were added and the reaction mixture stirred another 30 min. Subsequently the solid was collected by centrifugation (4000 rpm, 15 min) yielding 165 mg (75 %) **fC**. Although the product was pure according to NMR, it was subjected to HPLC purification (0-25 % MeCN in 45 min) before it was used as a standard for the mass spectrometry experiments.

^{1}H NMR (400 MHz, D_2O/MeOD) δ (ppm) = 9.56 (s, 1H), 8.85 (s, 1H), 6.24 (t, ^3J=6.1, 1H), 4.48 (dt, ^3J=4.8, ^3J=6.5, 1H), 4.18 (dd, ^3J=4.6, ^3J=8.0, 1H), 3.96 (dd, ^3J=3.4, ^2J=12.6, 1H), 3.84 (dd, ^3J=4.9, ^2J=12.6, 1H), 2.62 (ddd, ^3J=4.9, ^3J=6.5, ^2J=14.1, 1H), 2.46 – 2.37 (m, 1H). ^{13}C NMR (101 MHz, D_2O/MeOD) δ (ppm) = 190.3, 162.6, 155.2, 154.6, 105.7, 87.4, 87.1, 69.7, 60.6, 40.0. HRMS (ESI+): calculated for $C_{10}H_{14}N_3O_5^+$ $[M+H]^+$: 256.0928, found: 256.0928.

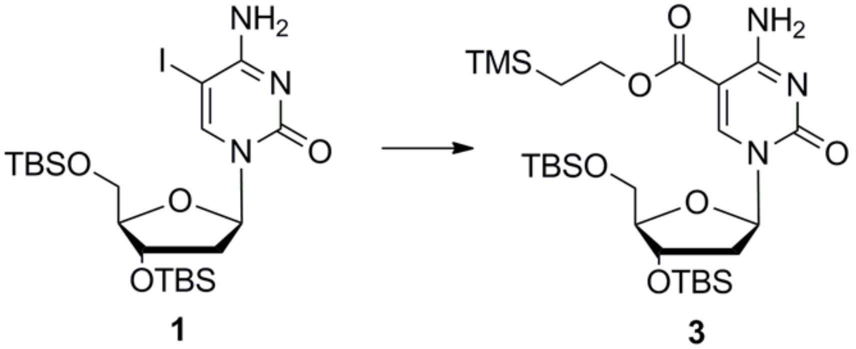

1 **3**

In a high pressure glass autoclave 1.00 g **1** (1.72 mmol, 1.0 eq), 49 mg $PdCl_2(MeCN)_2$ (0.19 mmol, 0.11 eq) and 600 µL DIPEA (3.44 mmol, 2.0 eq) were dissolved in 10 mL TMS-Et-OH. The autoclave was flushed with CO twice to remove residual air and subsequently the reaction stirred at a CO pressure of 3.5 bar at 60 °C for 24 h. After completion of the reaction the CO was discharged and the solvent evaporated *in vacuo*. The crude product was purified by column chromatography (*i*Hex/EtOAc 4:1→2:1) to yield 537 mg (52 %) **3** as a yellowish solid.

^{1}H NMR (599 MHz $CDCl_3$) δ (ppm) = 8.57 (s, 1H), 7.96 (s, 1H), 6.56 (s, 1H), 6.16 (dd, ^3J=5.9, ^3J=7.3, 1H), 4.40 – 4.32 (m, 2H), 4.28 – 4.22 (m, 1H), 4.06 (dd, ^3J=3.7, ^3J=6.2, 1H), 3.80 – 3.77 (m, 1H), 3.75 (dd, ^3J=2.5, ^2J=9.7, 1H), 2.63 (ddd, ^3J=2.3, ^3J=5.8, ^2J=13.5, 1H), 1.95 (ddd, ^3J=5.9, ^3J=7.4, ^2J=13.4, 1H), 1.09 – 1.05 (m, 2H), 0.88 (s, 9H), 0.86 (s, 9H), 0.07 (2x s, 18H), 0.05 (s, 3H). ^{13}C NMR (151 MHz, $CDCl_3$) δ (ppm) = 165.0, 163.7, 154.1, 147.3, 95.5, 88.8, 89.0, 72.9, 63.32, 63.30, 42.6, 25.8, 25.7, 18.2, 18.0, 17.7, -1.4, -1.6, -1.7, -4.7, -4.9, -5.5, -5.6. HRMS (ESI+): calculated for $C_{27}H_{54}N_3O_6Si_3^+$ $[M+H]^+$: 600.3315, found: 600.3319.

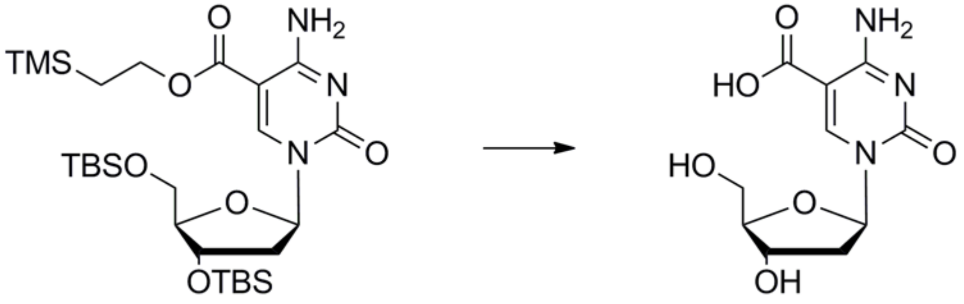

3 **caC**

In a flame dried round bottom flask 100 mg **3** (0.17 mmol, 1.0 eq) were dissolved in 10 mL DCM and 500 µL TBAF (1M in THF, 0.50 mmol, 3.0 eq) added. The solution was stirred at room temperature for 3 h. Subsequently 1 mL TMSOMe were added and the reaction mixture stirred for 30 min. The solvents were evaporated *in vacuo* and the crude product purified by HPLC (0-25 % MeCN in 45 min) to yield 32 mg (69 %) **caC** as a colorless solid.

^{1}H NMR (400 MHz, D_2O) δ (ppm) = 8.55 (s, 1H), 6.31 (t, ^3J=6.5, 1H), 4.51 (m, 1H), 4.13 (dd, ^3J=4.2, ^3J=8.1, 1H), 3.91 (dd, ^3J=3.5, ^2J=12.4, 1H), 3.83 (dd, ^3J=5.3, ^2J=12.5, 1H), 2.52 (ddd, ^3J=4.3, ^3J=6.4, ^2J=14.2, 1H), 2.41 – 2.32 (m, 1H). ^{13}C NMR (101 MHz, D_2O) δ (ppm) = 170.5, 164.6, 156.3, 146.3, 103.0, 86.8, 86.4, 70.4, 61.2, 39.6. HRMS (ESI+): calculated for $C_{10}H_{14}N_3O_6^+$ [M+H]$^+$: 272.0877, found: 272.0878.

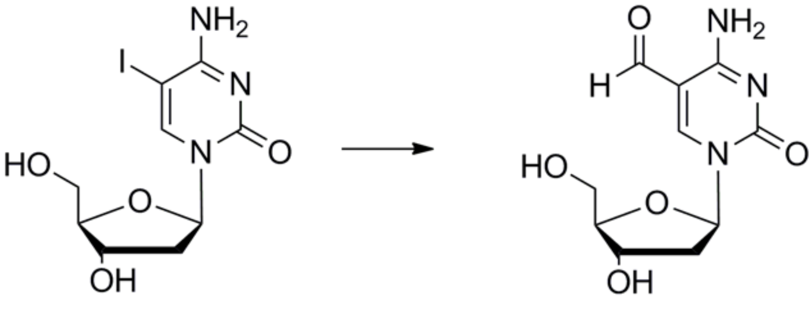

In a high pressure glass autoclave 100 mg **5** (0.28 mmol, 1.0 eq), 45 mg PPh_3 (0.17 mmol, 0.6 eq) and 29 mg $Pd_2(dba)_3$*$CHCl_3$ (0.03 mmol, 0.1 eq) were dissolved in 10 mL DMF. The autoclave was flushed with CO twice to remove residual air and subsequently the reaction stirred at a CO pressure of 3.5 bar at 60 °C. With a syringe pump 95 µL Bu_3SnH (0.34 mmol, 1.2 eq, dissolved in 900 µL toluene) were added through a septum at 0.1 mL per hour. After completion of the addition the reaction mixture was stirred additional 12 hours at 60 °C. Subsequently the CO was discharged and the solvent evaporated *in vacuo*. The crude product was purified by column chromatography (DCM/MeOH 9:1) to yield 25 mg (35 %) **fC** as a yellow solid.

^{1}H NMR (400 MHz, D_2O/MeOD) δ (ppm) = 9.56 (s, 1H), 8.85 (s, 1H), 6.24 (t, ^3J=6.1, 1H), 4.48 (dt, ^3J=4.8, ^3J=6.5, 1H), 4.18 (dd, ^3J=4.6, ^3J=8.0, 1H), 3.96 (dd, ^3J=3.4, ^2J=12.6, 1H), 3.84 (dd, ^3J=4.9, ^2J=12.6, 1H), 2.62 (ddd, ^3J=4.9, ^3J=6.5, ^2J=14.1, 1H), 2.46 – 2.37 (m, 1H). ^{13}C NMR (101 MHz, D_2O/MeOD) δ (ppm) = 190.3, 162.6, 155.2, 154.6, 105.7, 87.4, 87.1, 69.7, 60.6, 40.0. HRMS (ESI+): calculated for $C_{10}H_{14}N_3O_5^+$ [M+H]$^+$: 256.0928, found: 256.0928.

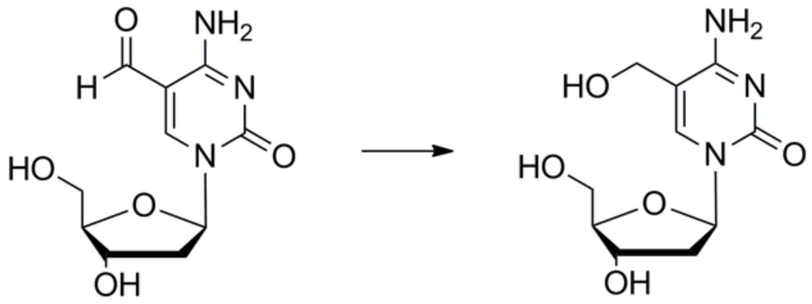

fC **hmC**

In a flame dried round bottom flask 16 mg **fC** (0.07 mmol, 1.0 eq) and 76 mg $CeCl_3$*7 H_2O (0.20 mmol, 3.0 eq) were dissolved in 3 mL methanol. To this solution 3 mg $NaBH_4$ (0.07 mmol, 1.0 eq) were added and the reaction mixture stirred at room temperature for 15 min. Subsequently the reaction mixture was evaporated to dryness and the crude product purified by reversed phase column chromatography (0-5% MeOH). Because the product contained a significant amount of salts it was desalted using a nucleoside desalting column (Waters Sep-Pak Plus Short tC18) to yield 9 mg **hmC** (53 %) as a colorless solid.

^{1}H NMR (400 MHz, D_2O/MeOD) δ (ppm) = 7.83 (s, 1H), 6.16 (t, ^3J=6.5, 1H), 4.42 – 4.25 (m, 3H), 3.95 (dd, ^3J=3.7, ^3J=8.5, 1H), 3.76 (dd, ^3J=3.4, ^2J=12.5, 1H), 3.67 (dd, ^3J=4.8, ^2J=12.5, 1H), 2.34 (ddd, ^3J=4.1, ^3J=6.4, ^2J=14.0, 1H), 2.24 – 2.11 (m, 1H). ^{13}C NMR (101 MHz, D_2O/MeOD) δ (ppm) = 166.1, 158.3, 141.3, 107.8, 88.0, 87.3, 71.6, 62.3, 59.0, 41.0. HRMS (ESI-): calculated for $C_{10}H_{14}N_3O_5^-$ [M-H]$^-$: 256.0939, found: 256.0936.

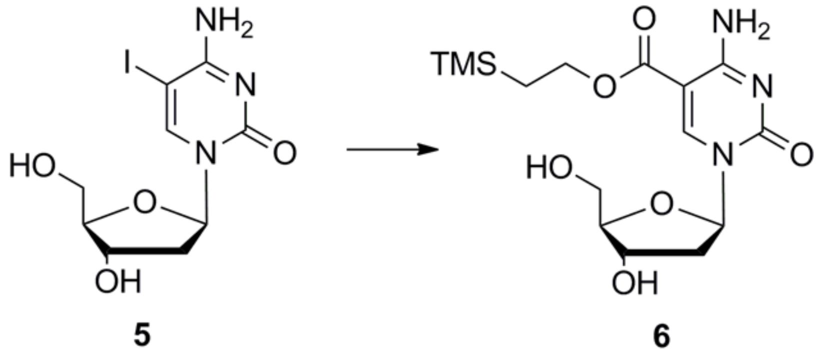

5 **6**

In a high pressure glass autoclave 300 mg **5** (0.85 mmol, 1.0 eq), 24 mg $PdCl_2(MeCN)_2$ (0.09 mmol, 0.11 eq) and 296 µL DIPEA (1.70 mmol, 2.0 eq) were

dissolved in 10 mL TMS-Et-OH. The autoclave was flushed with CO twice to remove residual air and subsequently the reaction stirred at a CO pressure of 3.5 bar at 60 °C for 24 h. After completion of the reaction the CO was discharged and the solvent evaporated *in vacuo*. The crude product was purified by column chromatography (DCM/MeOH 19:1) to yield 70 mg (22 %) **6** as a colorless oil.

^{1}H NMR (400 MHz, MeOD) δ (ppm) = 9.05 (s, 1H), 6.19 (t, ^{3}J=6.1, 1H), 4.42 – 4.32 (m, 3H), 4.00 (q, ^{3}J=3.5, 1H), 3.84 (dd, ^{3}J=3.4, ^{2}J=11.9, 1H), 3.76 (dd, ^{3}J=3.5, ^{2}J=12.0, 1H), 2.48 (ddd, ^{3}J=4.6, ^{3}J=6.3, ^{2}J=13.7, 1H), 2.20 (dt, ^{3}J=6.1, ^{2v}J=13.7, 1H), 1.13 (ddd, ^{3}J=1.2, ^{3}J=6.7, ^{2}J=8.6, 2H), 0.08 (s, 9H). ^{13}C NMR (101 MHz, MeOD) δ (ppm) = 166.4, 165.4, 156.7, 149.9, 97.5, 89.4, 88.7, 71.7, 64.6, 62.4, 42.80, 18.36, -1.32. HRMS (ESI+): calculated for $C_{15}H_{26}N_3O_6Si^+$ [M+H]$^+$: 372.1585, found: 372.1587.

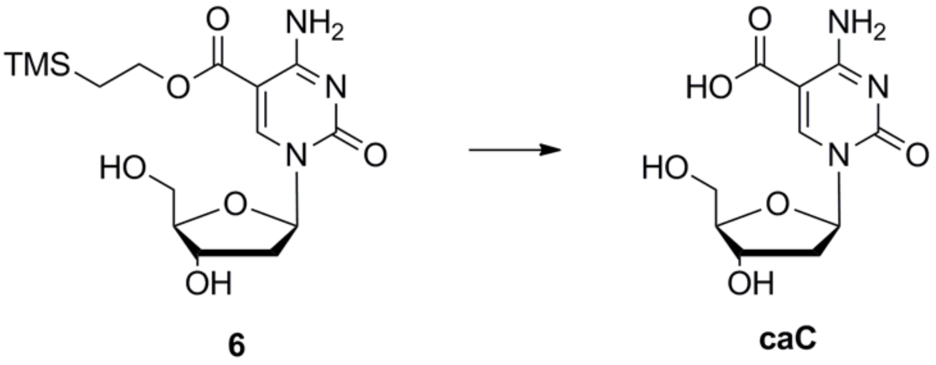

In a polypropylene tube 70 mg **6** (0.19 mmol, 1.0 eq) were dissolved in 7 mL DCM, subsequently 76 µL pyridine (0.94 mmol, 5.0 eq) and 73 µL HF*pyridine (70 % HF, 2.83 mmol, 15.0 eq) were added and the reaction mixture stirred 14 h at room temperature. 500 µL TMSOMe were added and the reaction mixture stirred another 30 min, diluted with DCM and extracted with 20 mL H_2O. The aqueous layer was evaporated to dryness and the crude product purified by HPLC (0-25 % MeCN in 45 min) yielding 7 mg (11 %) **caC**.

^{1}H NMR (400 MHz, D_2O) δ (ppm) = 8.55 (s, 1H), 6.31 (t, ^{3}J=6.5, 1H), 4.51 (m, 1H), 4.13 (dd, ^{3}J=4.2, ^{3}J=8.1, 1H), 3.91 (dd, ^{3}J=3.5, ^{2}J=12.4, 1H), 3.83 (dd, ^{3}J=5.3, ^{2}J=12.5, 1H), 2.52 (ddd, ^{3}J=4.3, ^{3}J=6.4, ^{2}J=14.2, 1H), 2.41 – 2.32 (m, 1H). ^{13}C NMR (101 MHz, D_2O) δ (ppm) = 170.5, 164.6, 156.3, 146.3, 103.0, 86.8, 86.4, 70.4, 61.2, 39.6. HRMS (ESI+): calculated for $C_{10}H_{14}N_3O_6^+$ [M+H]$^+$: 272.0877, found: 272.0878.

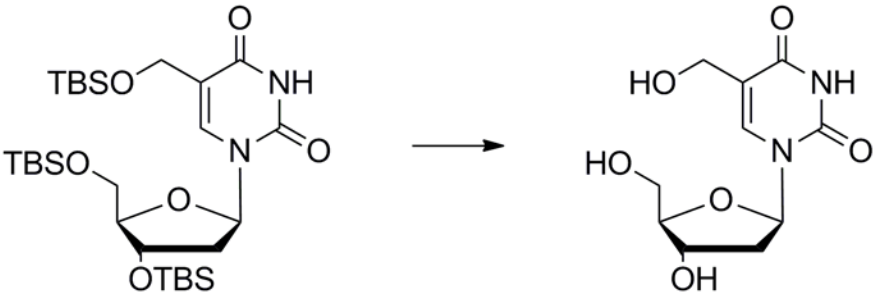

4 hmU

In a polypropylene tube 143 mg **4** (0.25 mmol, 1.0 eq) were dissolved in 14 mL EtOAc, subsequently 149 µL pyridine (1.85 mmol, 7.5 eq) and 141 µL HF*pyridine (70 % HF, 5.41 mml, 22.0 eq) were added and the reaction mixture stirred 14 h at room temperature. During this time a white solid crashed out. 1 mL TMSOMe were added and the reaction mixture stirred another 30 min. Subsequently the solid was collected by centrifugation (4000 rpm, 15 min). The product was subjected to HPLC purification (0-25 % MeCN in 45 min) to yield 43 mg **hmU** (70 %).

^{1}H NMR (400 MHz, D_2O) δ (ppm) = 7.87 (d, ^{4}J=0.7, 1H), 6.28 (t, ^{3}J=6.7, 1H), 4.45 (dt, ^{3}J=4.0, ^{3}J=7.6, 1H), 4.36 (d, ^{4}J=0.6, 2H), 4.02 (dd, ^{3}J=4.0, ^{3}J=7.9, 1H), 3.84 (dd, ^{3}J=3.5, ^{2}J=12.4, 1H), 3.76 (dd, ^{3}J=4.8, ^{2}J=12.4, 1H), 2.44 – 2.29 (m, 2H). ^{13}C NMR (101 MHz, D_2O) δ (ppm) = 167.0, 153.4, 140.3, 114.8, 87.8, 86.6, 71.6, 62.3, 57.8, 40.06. HRMS (ESI-): calculated for $C_{10}H_{13}N_2O_6^-$ [M-H]$^-$: 257.0779, found: 257.0781.

Oligonucleotide Synthesis

was performed on an Expedite 8909 Nucleic Acid Synthesis System (*PerSeptive Biosystems*) using standard DNA synthesis conditions. Phosphoramidites for hmdC, dA, dC, dG, dT and CPG carriers were obtained from *Glen Research*. The terminal DMT protecting group was kept on the oligonucleotides after synthesis and removed after cleavage from the resin (see Deprotection and purification). Except for hmC standard coupling conditions were used. For hmC coupling times were doubled to ensure good yields.

Deprotection and purification:

Deprotection and cleavage of the oligonucleotides from the CPG carrier was carried out in a mixture of saturated ammonia solution in water (7 M) and ethanol (3:1) at 60 °C for 60 hours. DNA purification was conducted on analytical and preparative HPLC (*Waters or Merck Hitachi*) using *Nucleosil* columns (250 * 4 mm, C18ec, particle size 3 µm or 250 * 10 mm, C18ec, 5 µm) from *Machery-Nagel*. The applied buffer was 0.1 M triethylammoniumacetate in water / 0.1 M triethylammoniumacetate in 80 % aqueous MeCN. The fractions were checked for purity by analytical HPLC and MALDI-MS. The purified oligonucletides were concentrated *in vacuo* using a *Savant Speed Vac.* The oligonucleotides still containing the trityl group were deprotected by addition of 100 µL of an 80% acetic acid solution. After incubation at r.t. for 20 min at room temperature 100 µL of water together with 60 µL of a 3 M solution of sodium acetate were added. The oligonucleotides were desalted with Sep-Pak® cartridges (*Waters*) prior to use.

The following sequence was used for the immunohistology-competition experiments:
5' GTA GCC AGG TCG CAC GCG TGC TA***X*** GAT GCG AGA CTG C 3' X = hmC

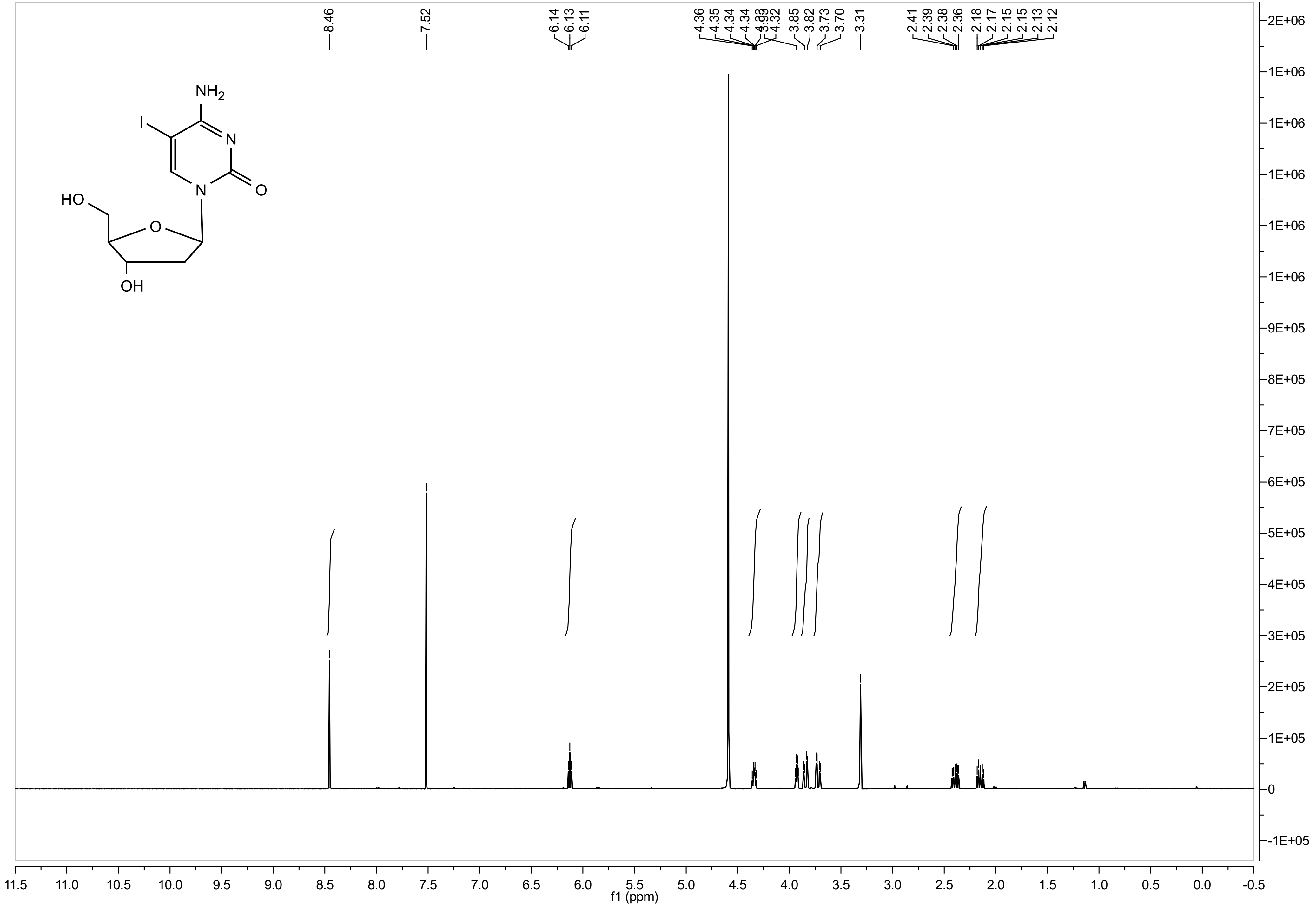

8.46
7.52
6.14
6.13
6.11
4.36
4.35
4.34
4.34
4.33
3.93
4.32
3.85
3.82
3.73
3.70
3.31
2.41
2.39
2.38
2.36
2.18
2.17
2.15
2.15
2.13
2.12
NH2
I
N
O
HO
O
N
OH
f1 (ppm)

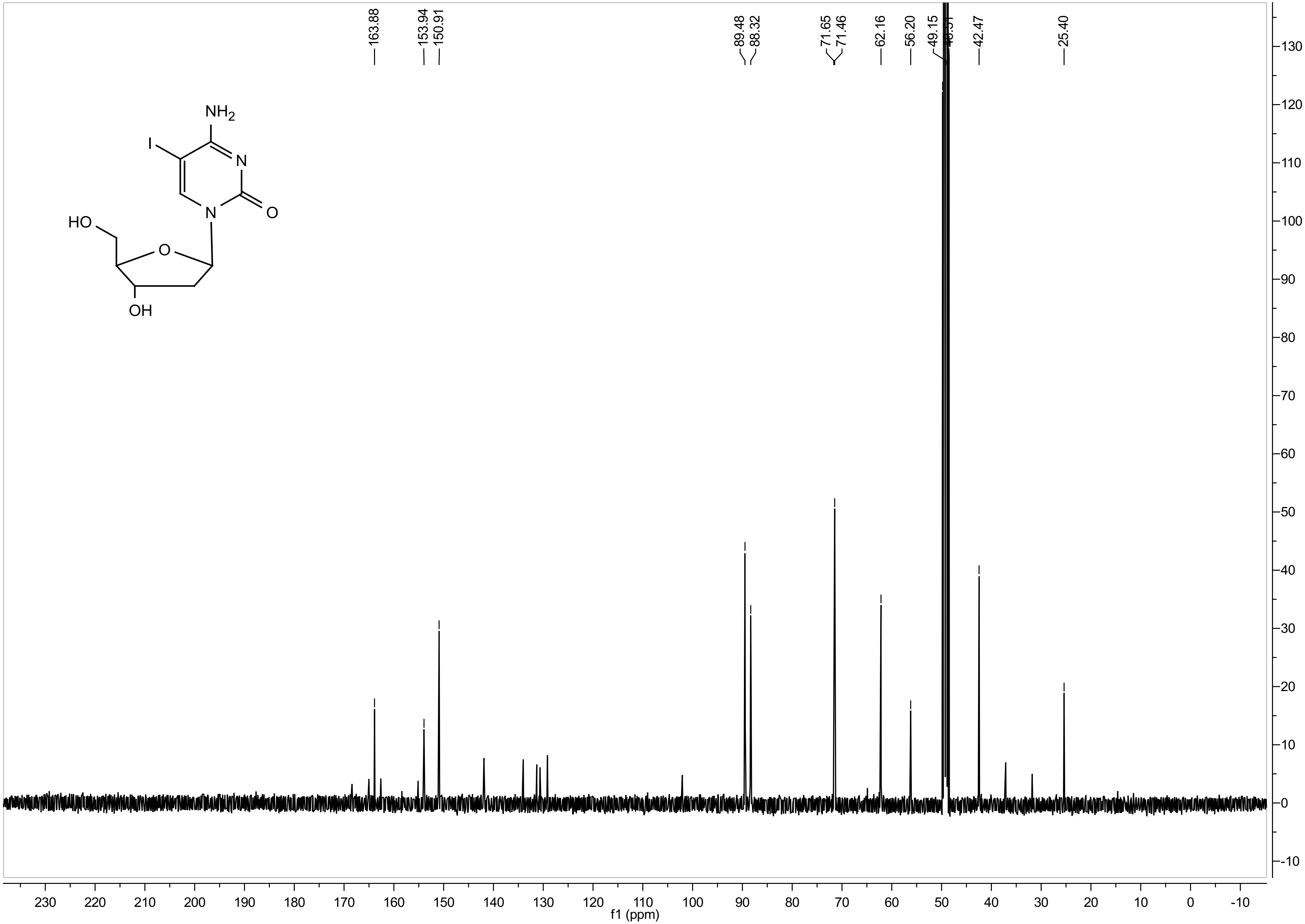

163.88
153.94
150.91
89.48
88.32
71.65
71.46
62.16
56.20
49.15
42.47
25.40
NH2
I
N
O
N
HO
O
OH
230 220 210 200 190 180 170 160 150 140 130 120 110 100 90 80 70 60 50 40 30 20 10 0 -10
f1 (ppm)
130 120 110 100 90 80 70 60 50 40 30 20 10 0 -10

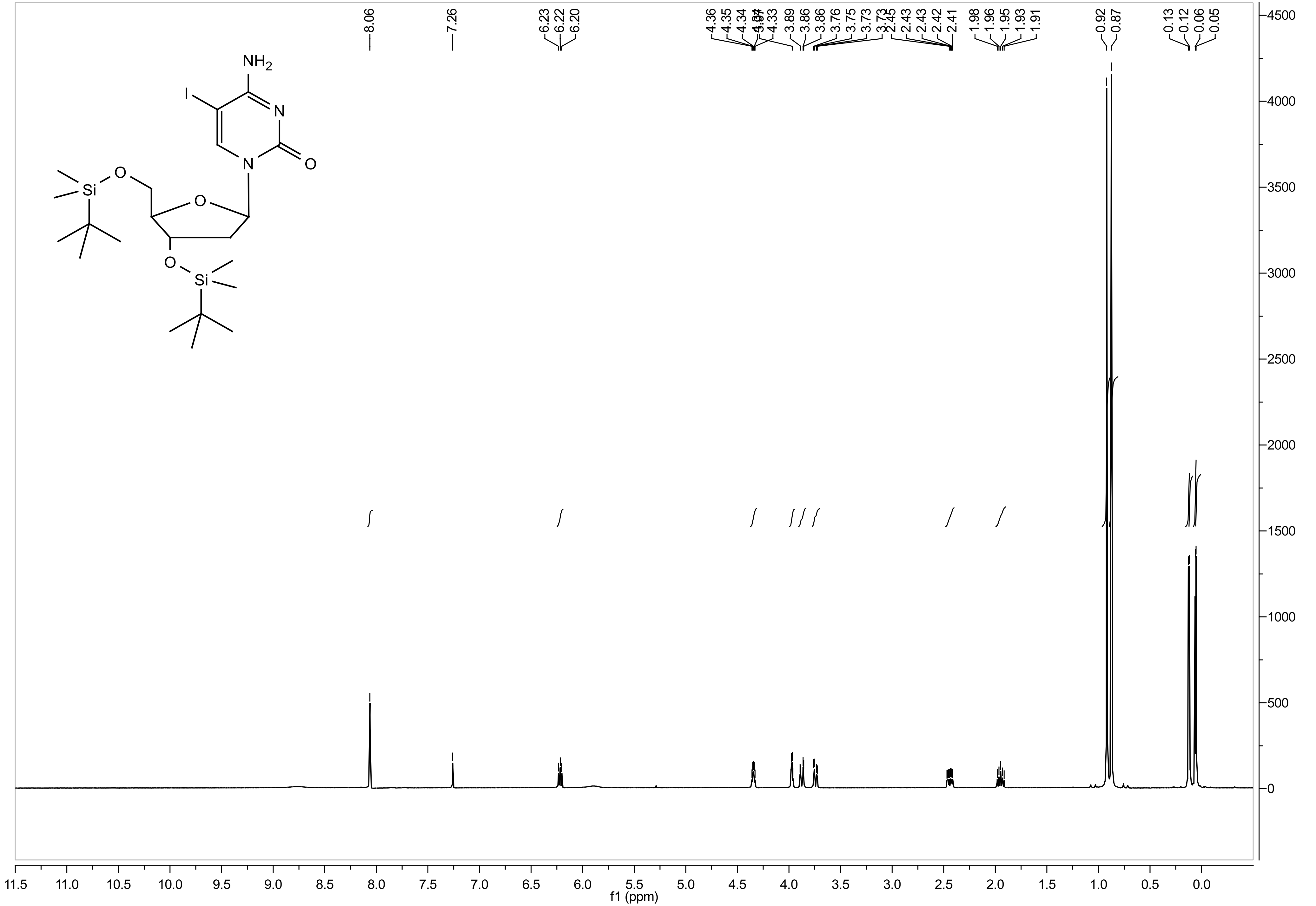

8.06
7.26
6.23
6.22
6.20
4.36
4.35
4.34
4.33
3.89
3.86
3.86
3.76
3.75
3.73
3.73
2.45
2.43
2.43
2.42
2.41
1.98
1.96
1.95
1.93
1.91
0.92
0.87
0.13
0.12
0.06
0.05
NH2
I
N
O
N
O
Si
O
O
Si
f1 (ppm)

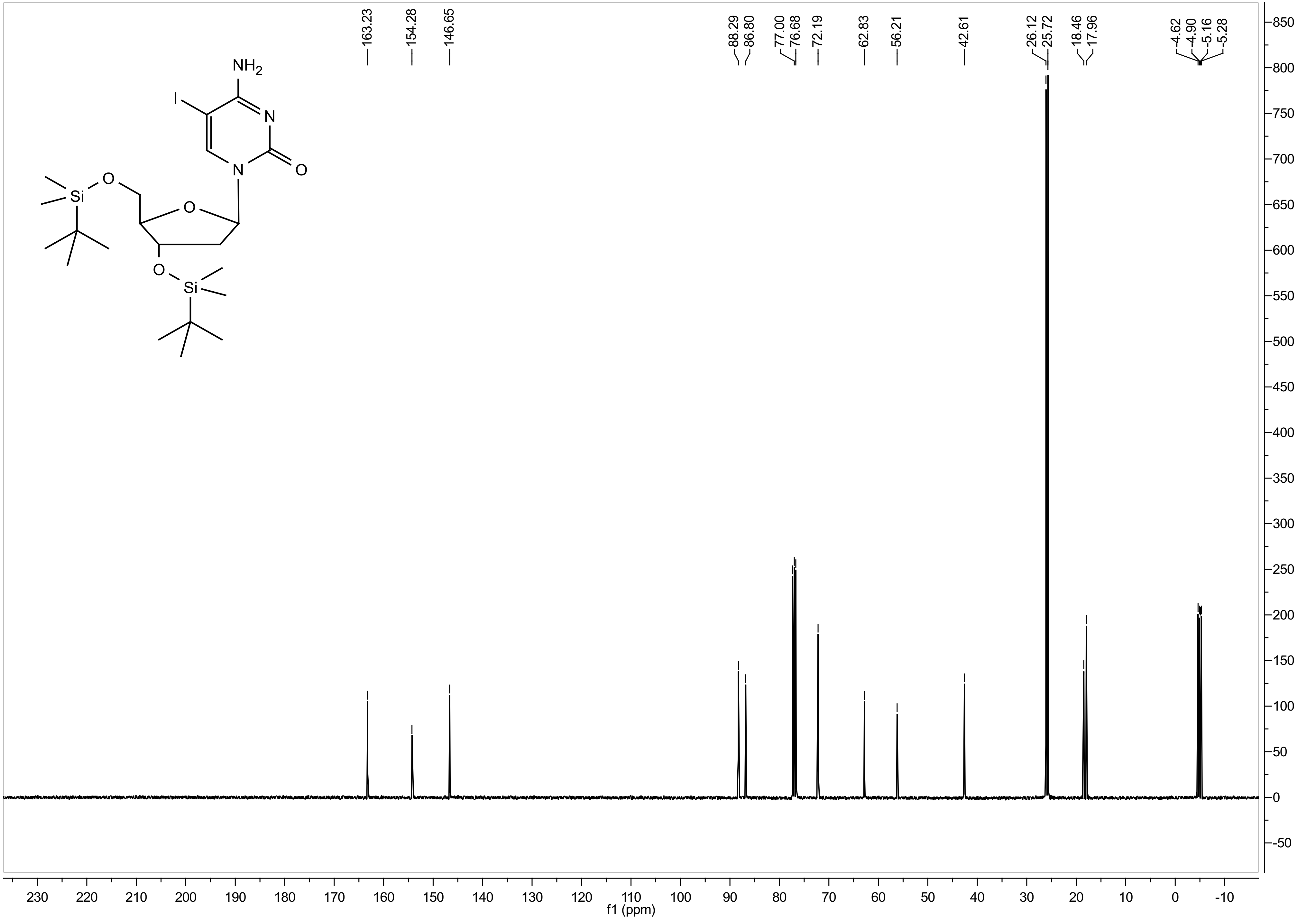

163.23
154.28
146.65
88.29
86.80
77.00
76.68
72.19
62.83
56.21
42.61
26.12
25.72
18.46
17.96
-4.62
-4.90
-5.16
-5.28
f1 (ppm)

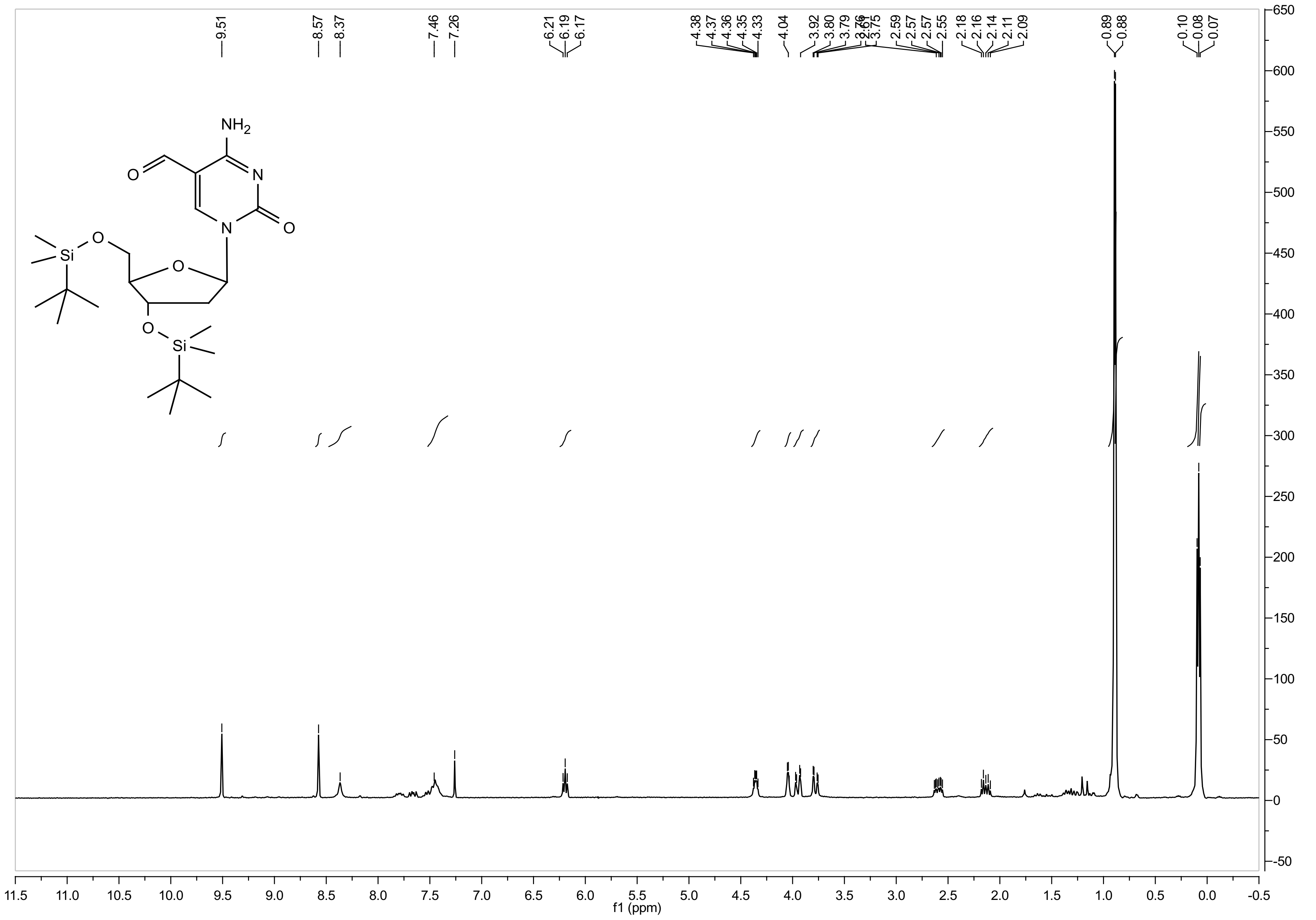

9.51
8.57
8.37
7.46
7.26
6.21
6.19
6.17
4.38
4.37
4.36
4.35
4.33
4.04
3.92
3.80
3.79
3.76
3.75
2.59
2.57
2.57
2.55
2.18
2.16
2.14
2.11
2.09
0.89
0.88
0.10
0.08
0.07
NH2
O
N
N
O
O
Si
O
O
Si
f1 (ppm)

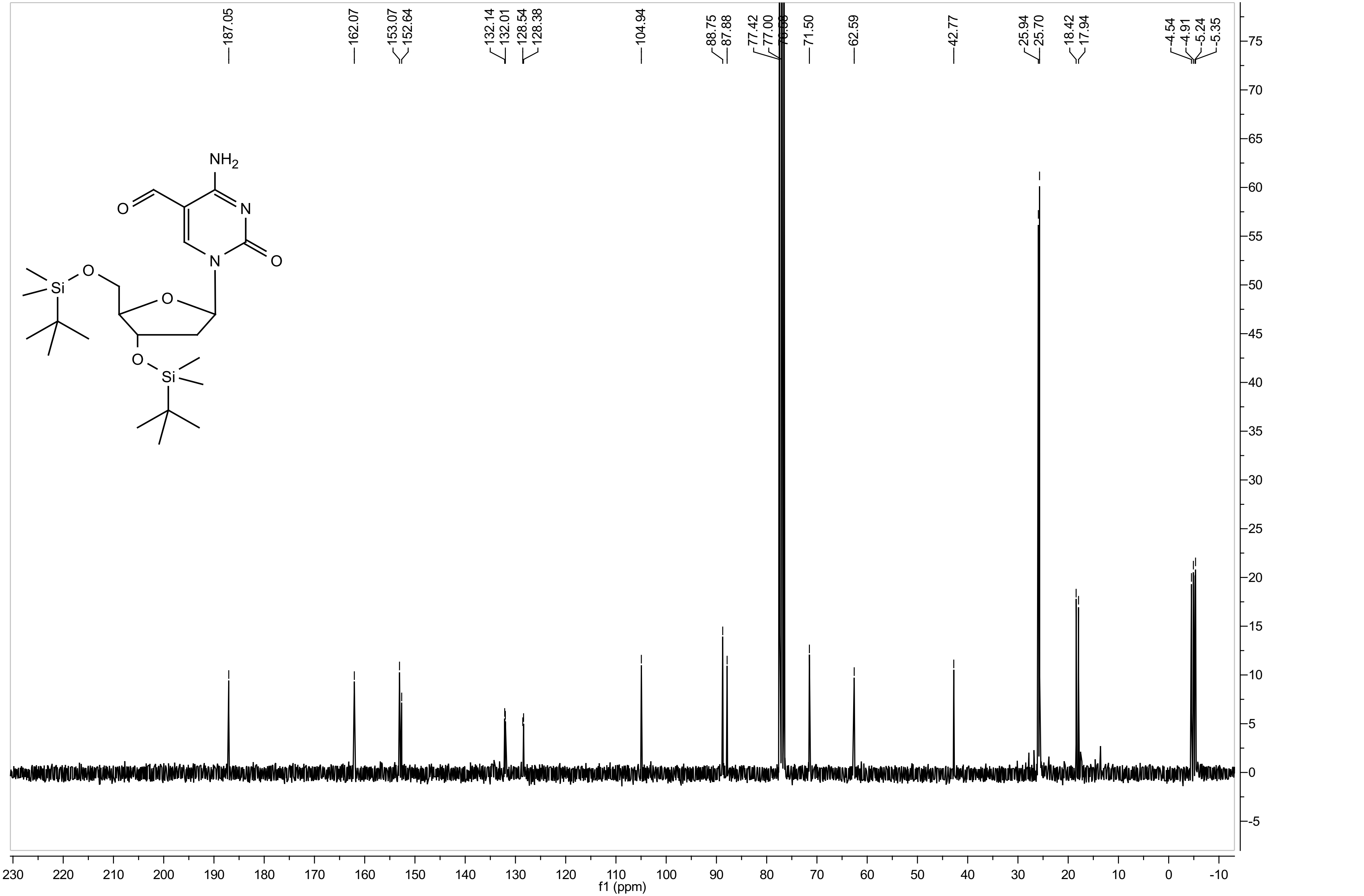
187.05
162.07
153.07
152.64
132.14
132.01
128.54
128.38
104.94
88.75
87.88
77.42
77.00
71.50
62.59
42.77
25.94
25.70
18.42
17.94
-4.54
-4.91
-5.24
-5.35
NH2
O
N
O
N
O
Si
O
O
Si
230 220 210 200 190 180 170 160 150 140 130 120 110 100 90 80 70 60 50 40 30 20 10 0 -10
f1 (ppm)
75 70 65 60 55 50 45 40 35 30 25 20 15 10 5 0 -5

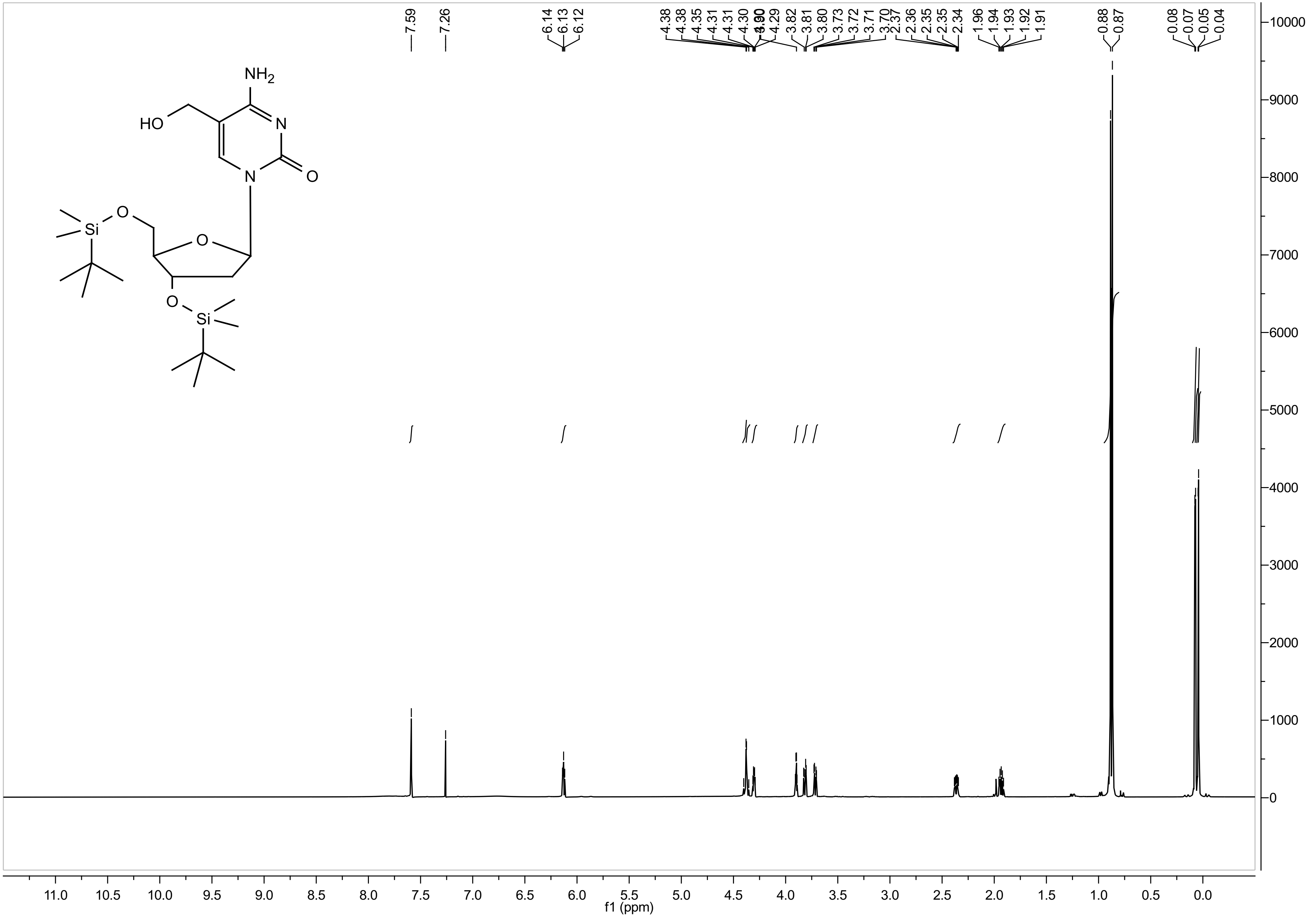
NH2
HO
N
O
N
O
Si
O
O
Si
7.59
7.26
6.14
6.13
6.12
4.38
4.38
4.35
4.31
4.31
4.30
4.29
3.82
3.81
3.80
3.73
3.72
3.71
3.70
2.37
2.36
2.35
2.35
2.34
1.96
1.94
1.93
1.92
1.91
0.88
0.87
0.08
0.07
0.05
0.04
10000
9000
8000
7000
6000
5000
4000
3000
2000
1000
0
11.0
10.5
10.0
9.5
9.0
8.5
8.0
7.5
7.0
6.5
6.0
5.5
5.0
4.5
4.0
3.5
3.0
2.5
2.0
1.5
1.0
0.5
0.0
f1 (ppm)

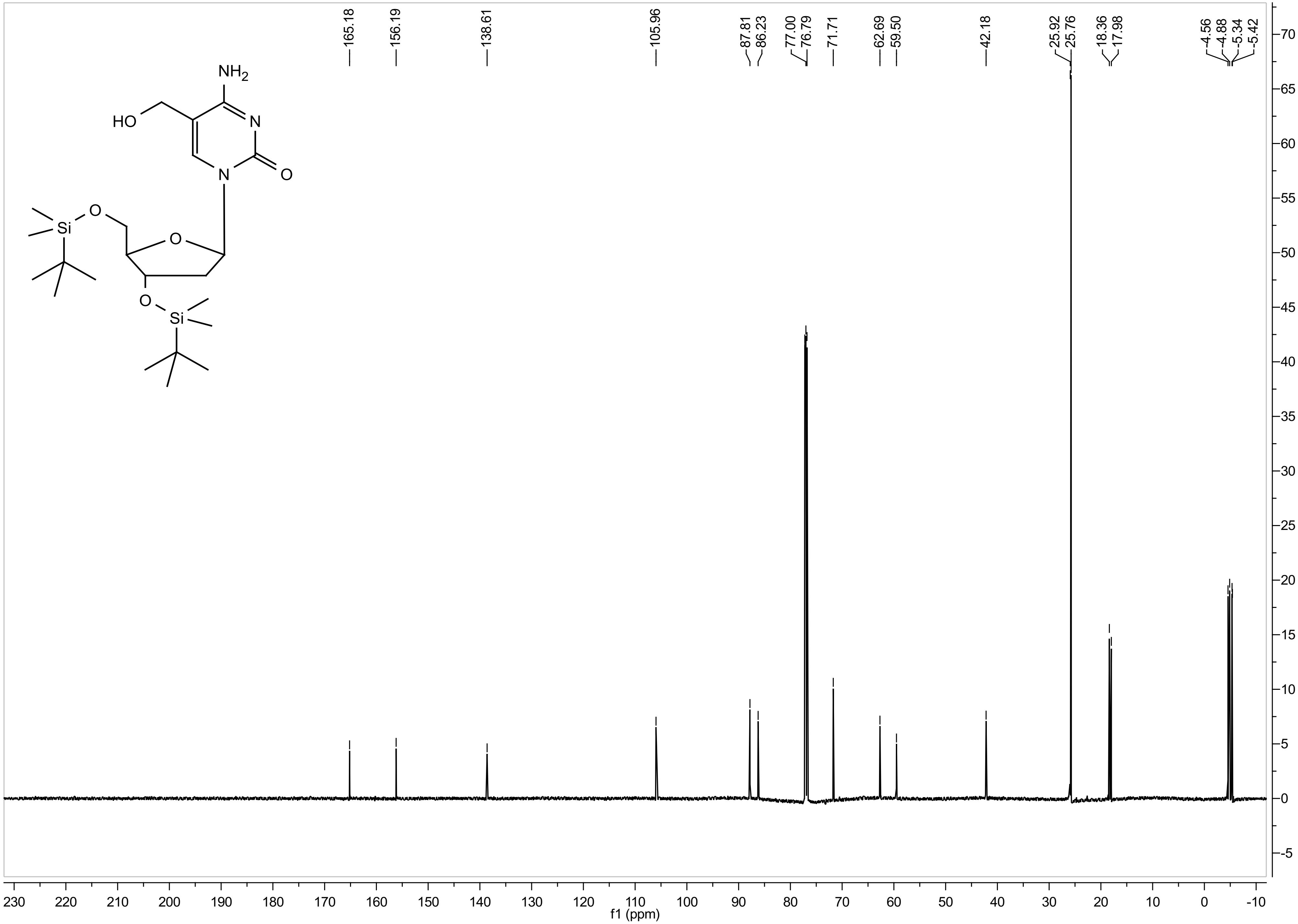

165.18
156.19
138.61
105.96
87.81
86.23
77.00
76.79
71.71
62.69
59.50
42.18
25.92
25.76
18.36
17.98
-4.56
-4.88
-5.34
-5.42
NH2
HO
N
O
Si
f1 (ppm)

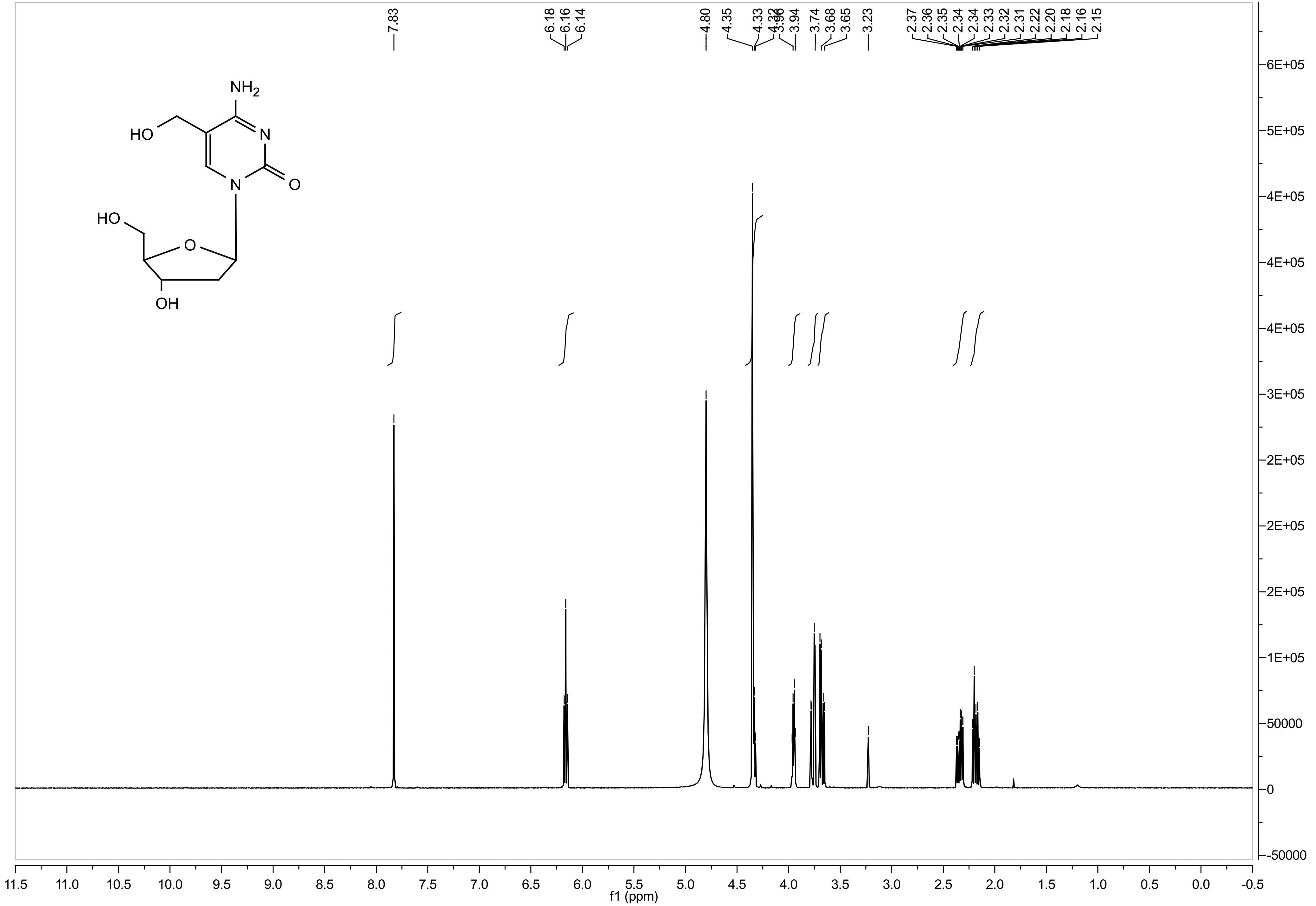

7.83
6.18
6.16
6.14
4.80
4.35
4.33
3.96
3.94
3.74
3.68
3.65
3.23
2.37
2.36
2.35
2.34
2.34
2.33
2.32
2.31
2.22
2.20
2.18
2.16
2.15
NH2
HO
N
O
N
HO
O
OH
f1 (ppm)

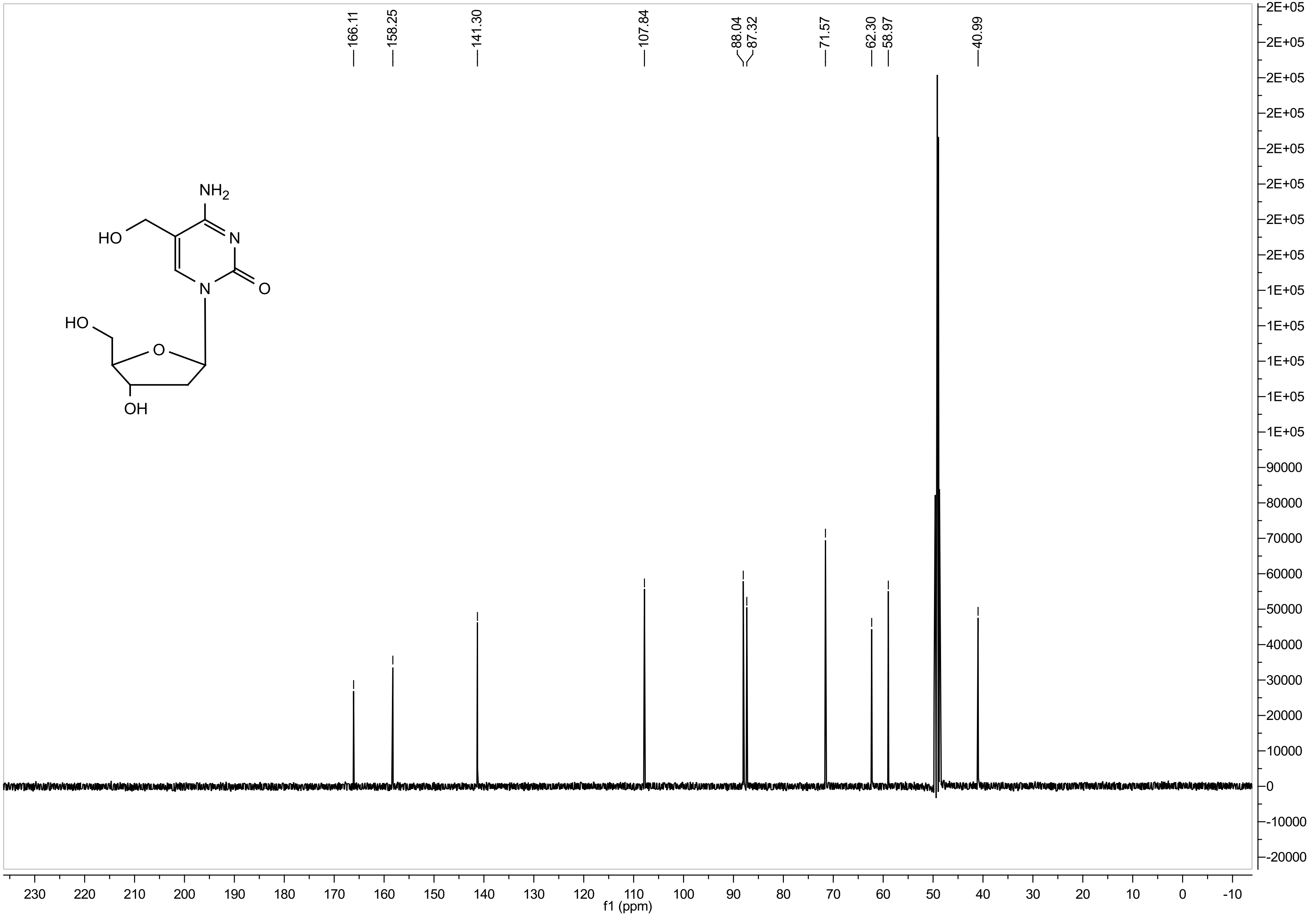

166.11
158.25
141.30
107.84
88.04
87.32
71.57
62.30
58.97
40.99
NH2
HO
N
O
N
HO
O
OH
f1 (ppm)

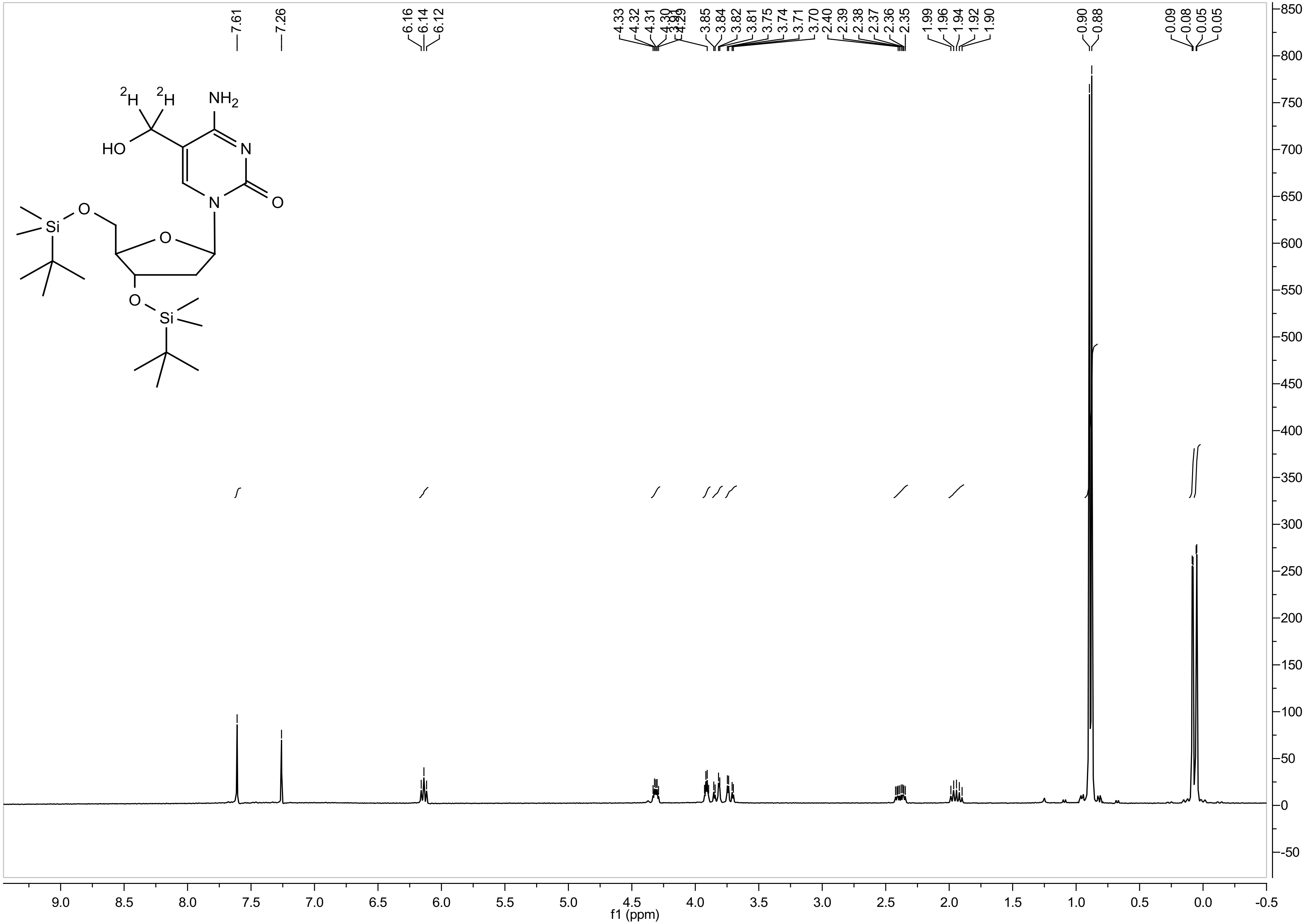
7.61
7.26
6.16
6.14
6.12
4.33
4.32
4.31
4.30
4.29
3.85
3.84
3.82
3.81
3.75
3.74
3.71
3.70
2.40
2.39
2.38
2.37
2.36
2.35
1.99
1.96
1.94
1.92
1.90
0.90
0.88
0.09
0.08
0.05
0.05
NH2
HO
N
O
Si
f1 (ppm)

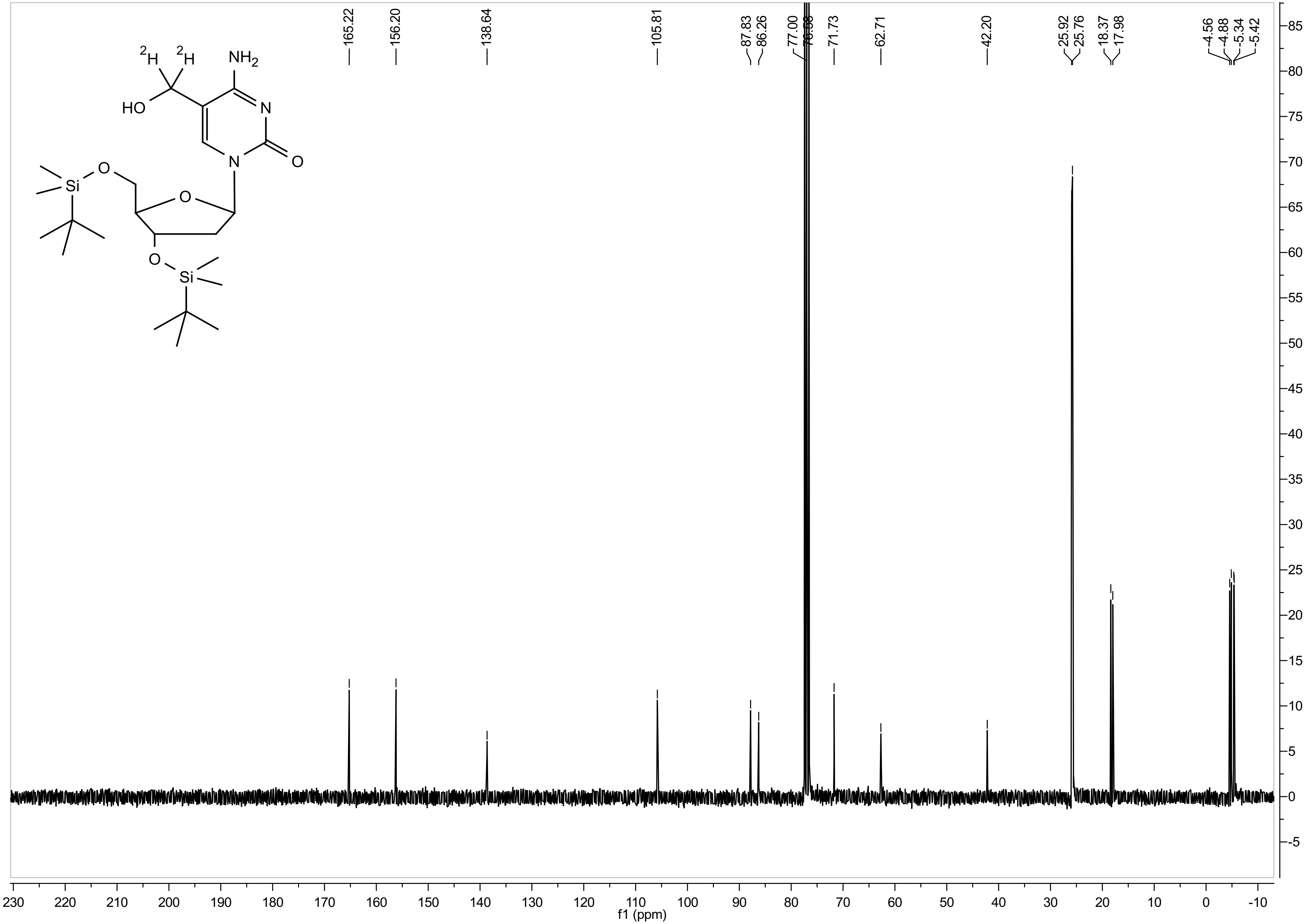

165.22
156.20
138.64
105.81
87.83
86.26
77.00
76.68
71.73
62.71
42.20
25.92
25.76
18.37
17.98
-4.56
-4.88
-5.34
-5.42
f1 (ppm)

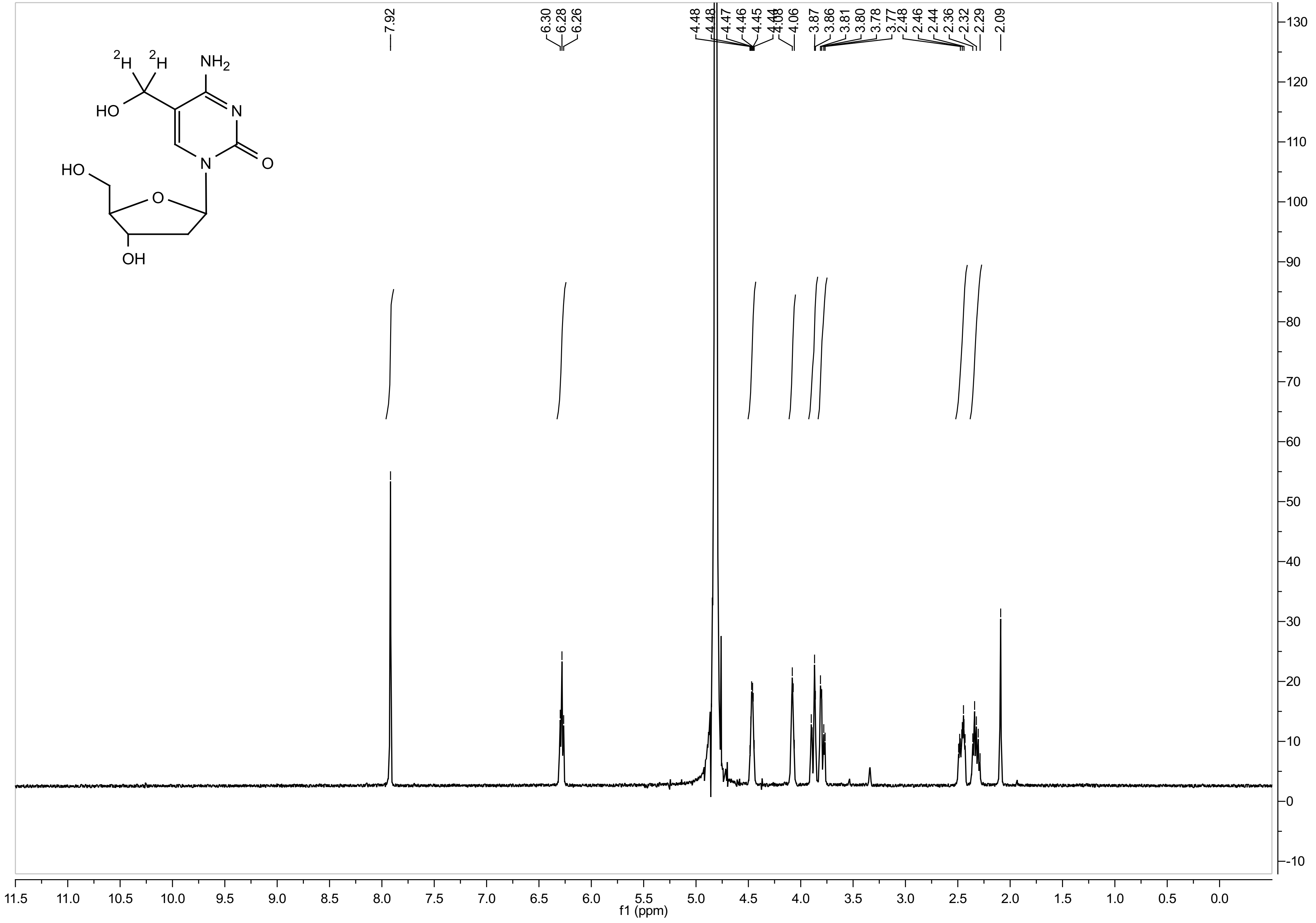
7.92
6.30
6.28
6.26
4.48
4.48
4.47
4.46
4.45
4.44
4.08
4.06
3.87
3.86
3.81
3.80
3.78
3.77
2.48
2.46
2.44
2.36
2.32
2.29
2.09
f1 (ppm)

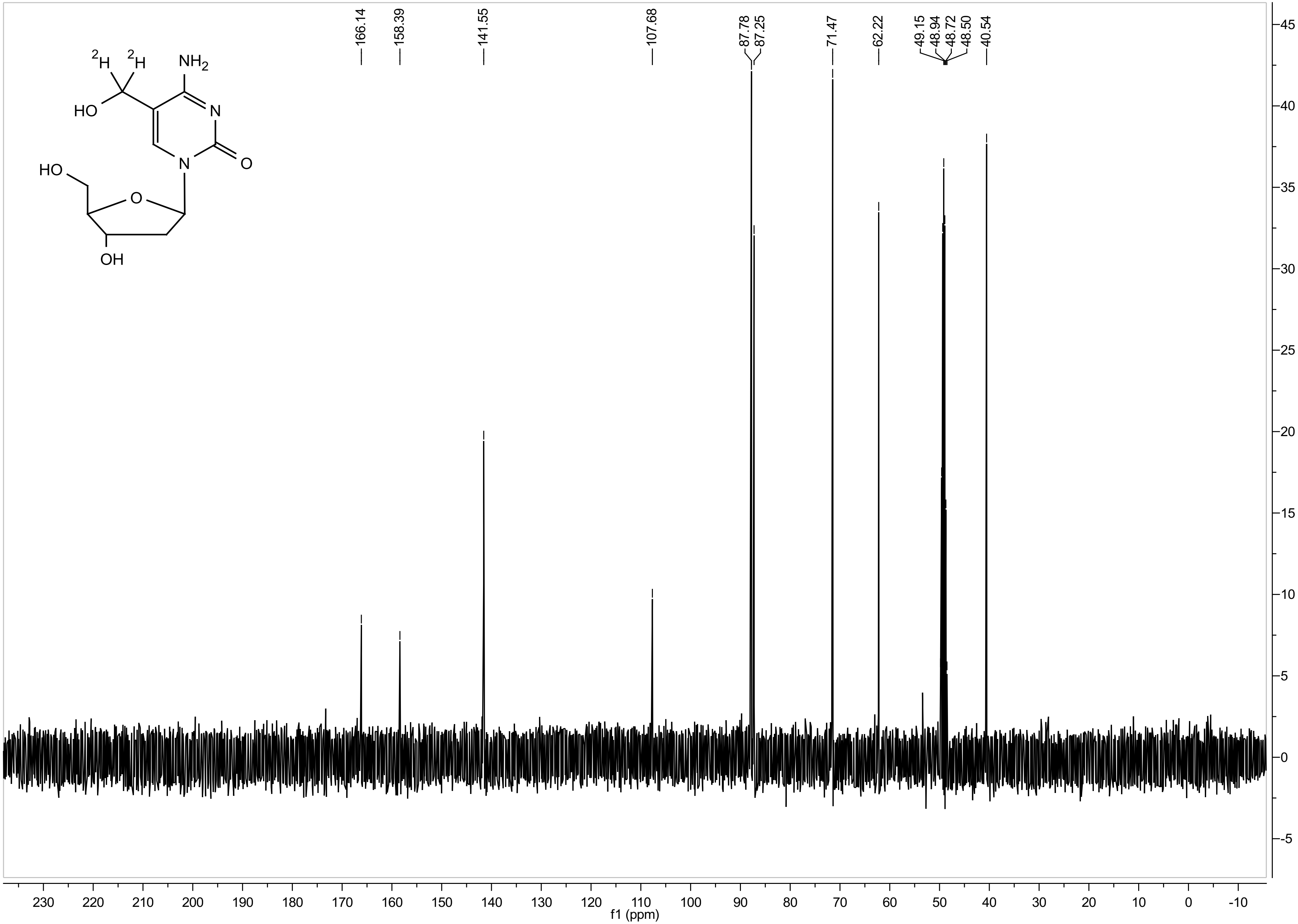

166.14
158.39
141.55
107.68
87.78
87.25
71.47
62.22
49.15
48.94
48.72
48.50
40.54
f1 (ppm)
HO
OH
NH2
N
O

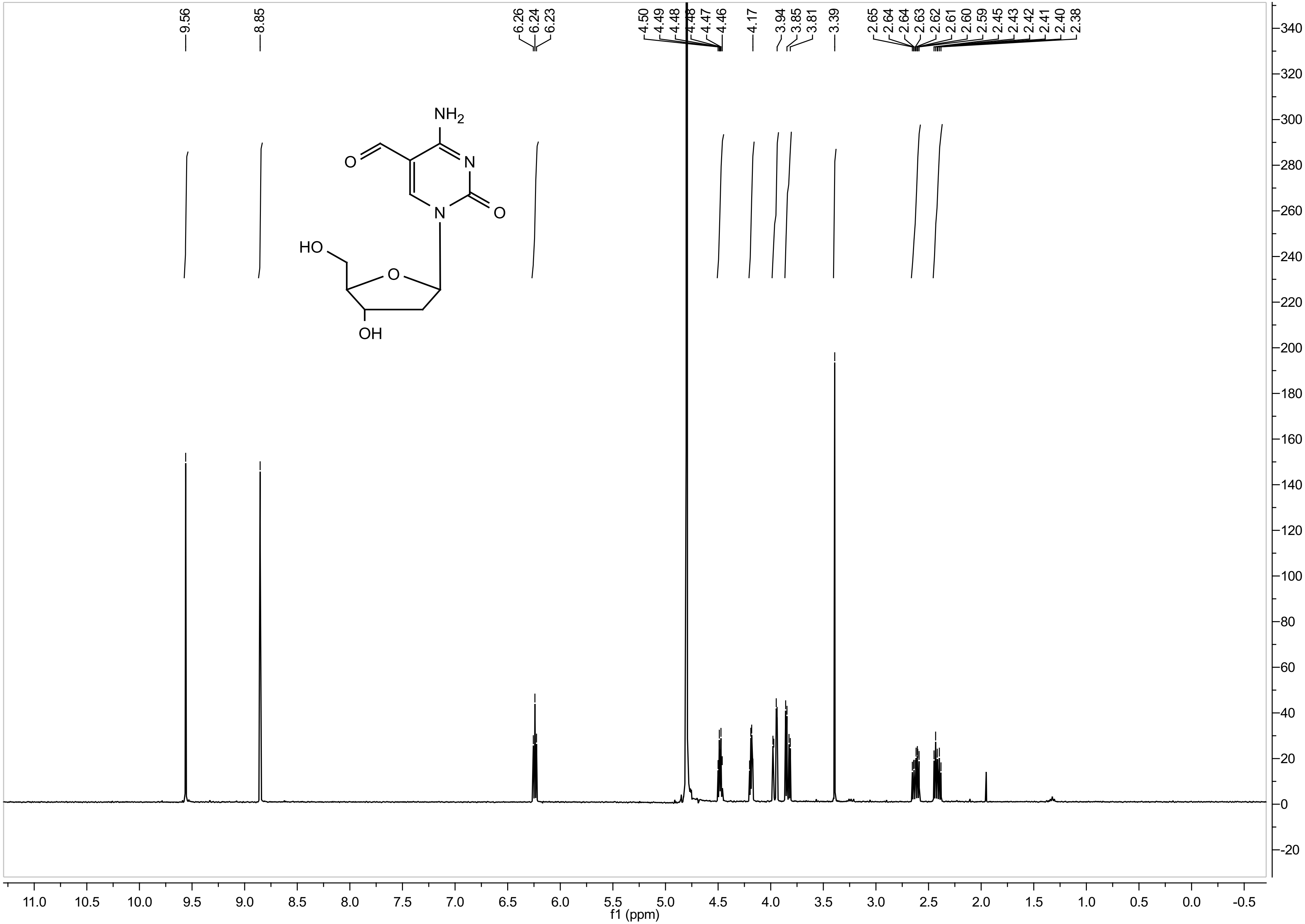
9.56
8.85
6.26
6.24
6.23
4.50
4.49
4.48
4.48
4.47
4.46
4.17
3.94
3.85
3.81
3.39
2.65
2.64
2.64
2.63
2.62
2.61
2.60
2.59
2.45
2.43
2.42
2.41
2.40
2.38
NH2
N
O
HO
OH
f1 (ppm)

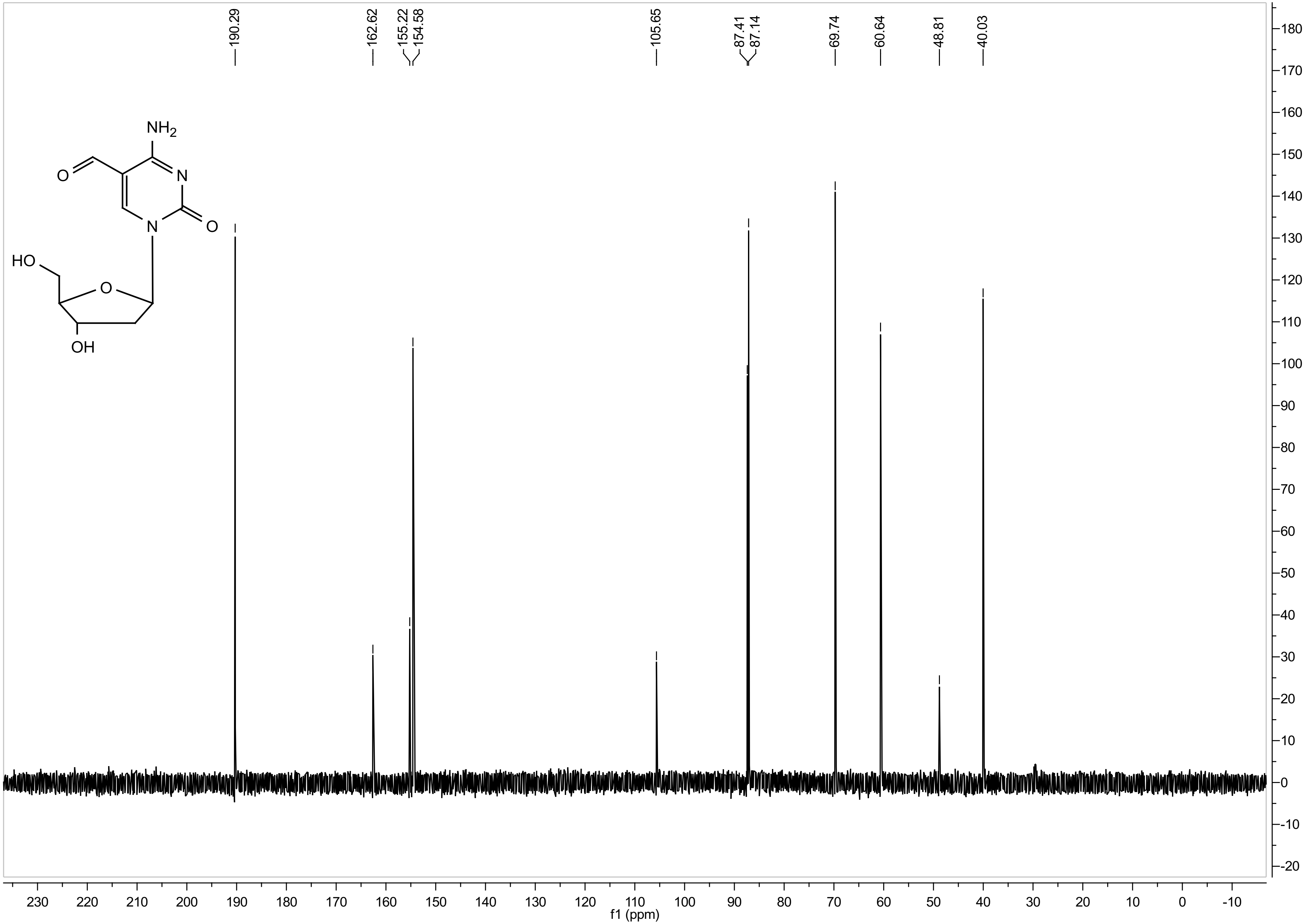

190.29
162.62
155.22
154.58
105.65
87.41
87.14
69.74
60.64
48.81
40.03
NH2
O
N
N
O
HO
O
OH
f1 (ppm)

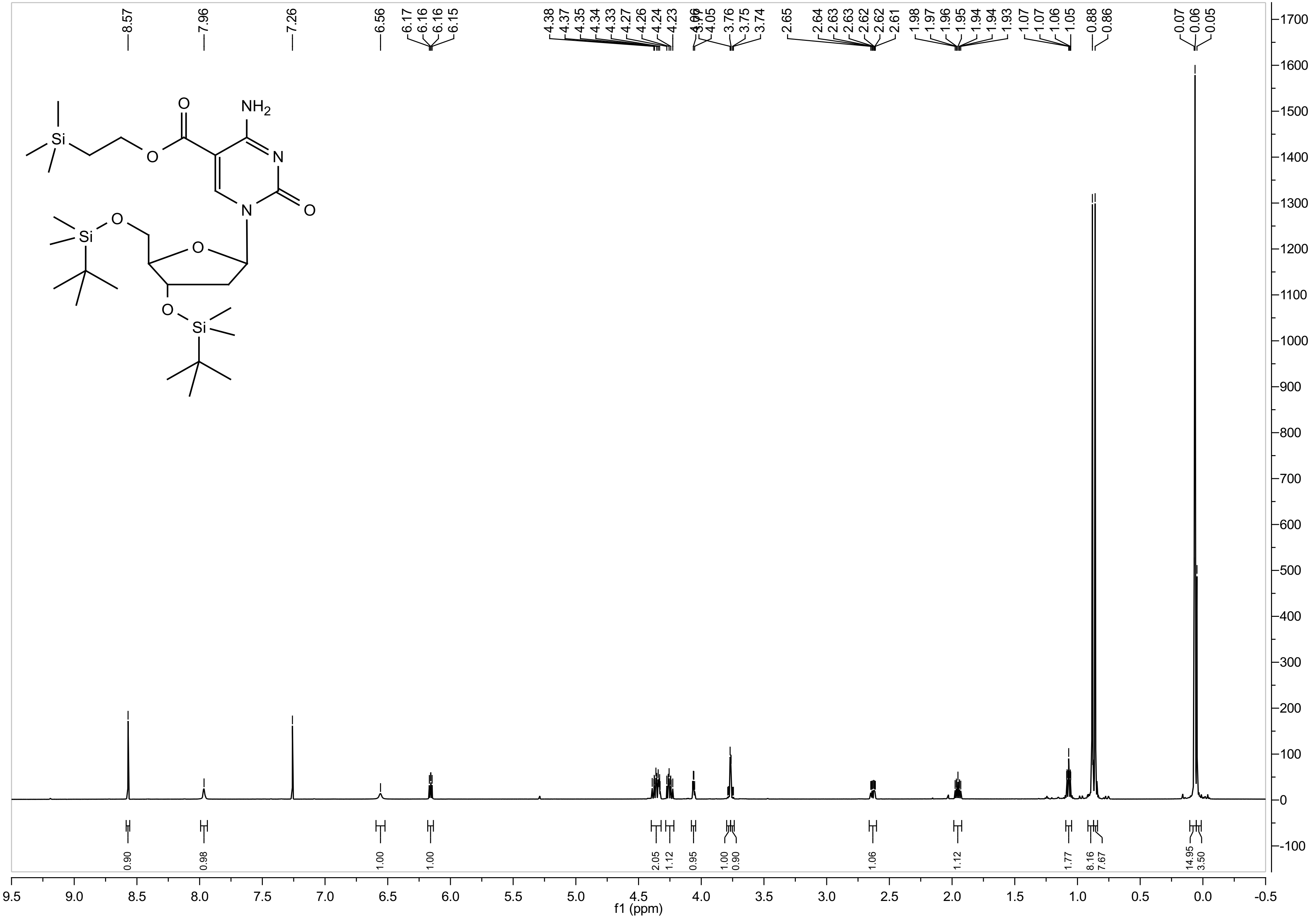
O
NH2
N
O
Si
O
N
O
Si
O
O
Si
8.57
7.96
7.26
6.56
6.17
6.16
6.16
6.15
4.38
4.37
4.35
4.34
4.33
4.27
4.26
4.24
4.23
4.05
3.76
3.75
3.74
2.65
2.64
2.63
2.63
2.62
2.62
2.61
1.98
1.97
1.96
1.95
1.94
1.94
1.93
1.07
1.07
1.06
1.05
0.88
0.86
0.07
0.06
0.05
0.90
0.98
1.00
1.00
2.05
1.12
0.95
1.00
0.90
1.06
1.12
1.77
8.16
7.67
14.95
3.50
9.5
9.0
8.5
8.0
7.5
7.0
6.5
6.0
5.5
5.0
4.5
4.0
3.5
3.0
2.5
2.0
1.5
1.0
0.5
0.0
-0.5
f1 (ppm)
1700
1600
1500
1400
1300
1200
1100
1000
900
800
700
600
500
400
300
200
100
0
-100

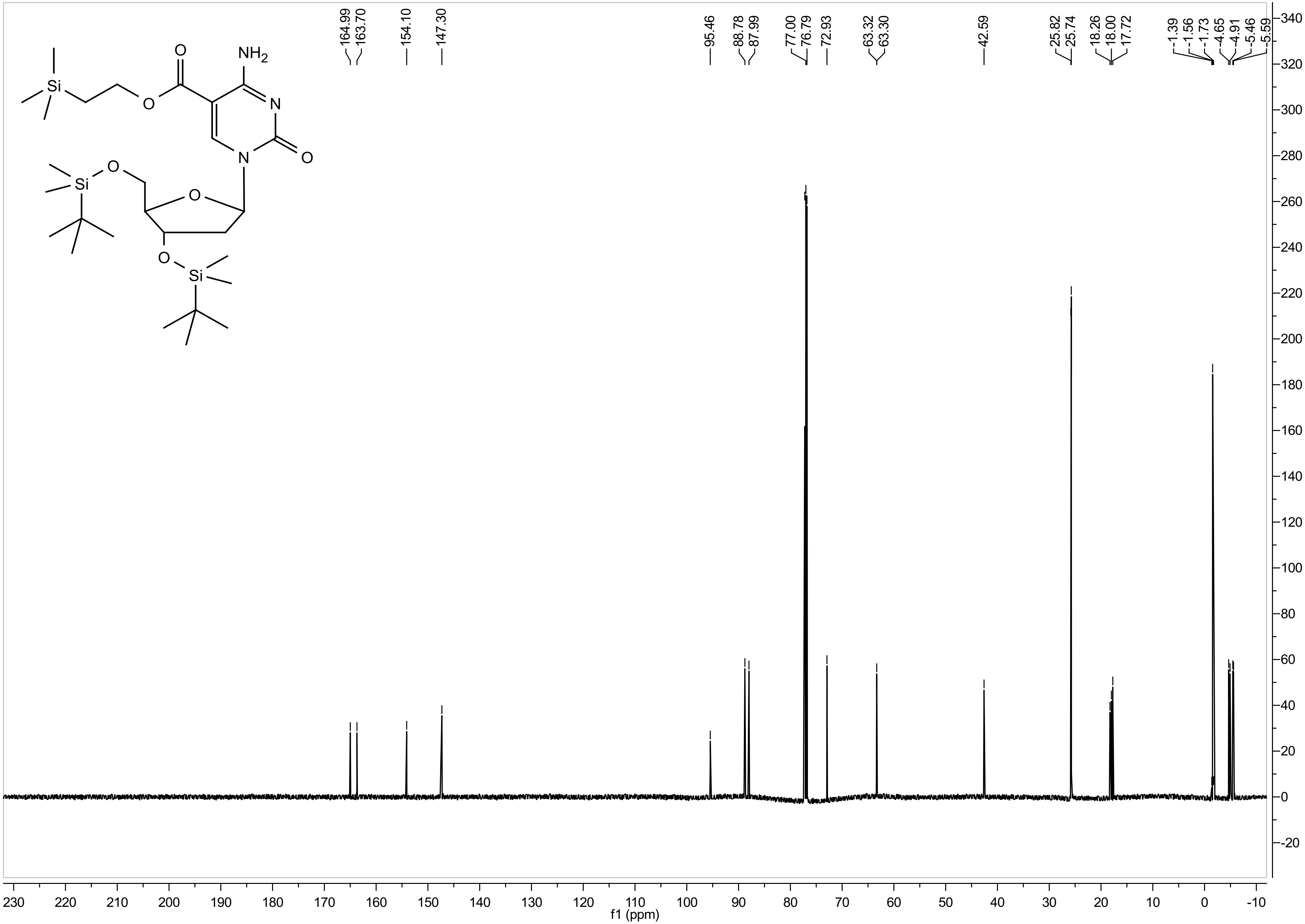
164.99
163.70
154.10
147.30
95.46
88.78
87.99
77.00
76.79
72.93
63.32
63.30
42.59
25.82
25.74
18.26
18.00
17.72
-1.39
-1.56
-1.73
-4.65
-4.91
-5.46
-5.59
NH2
O
N
O
N
O
O
Si
O
Si
O
O
Si
f1 (ppm)

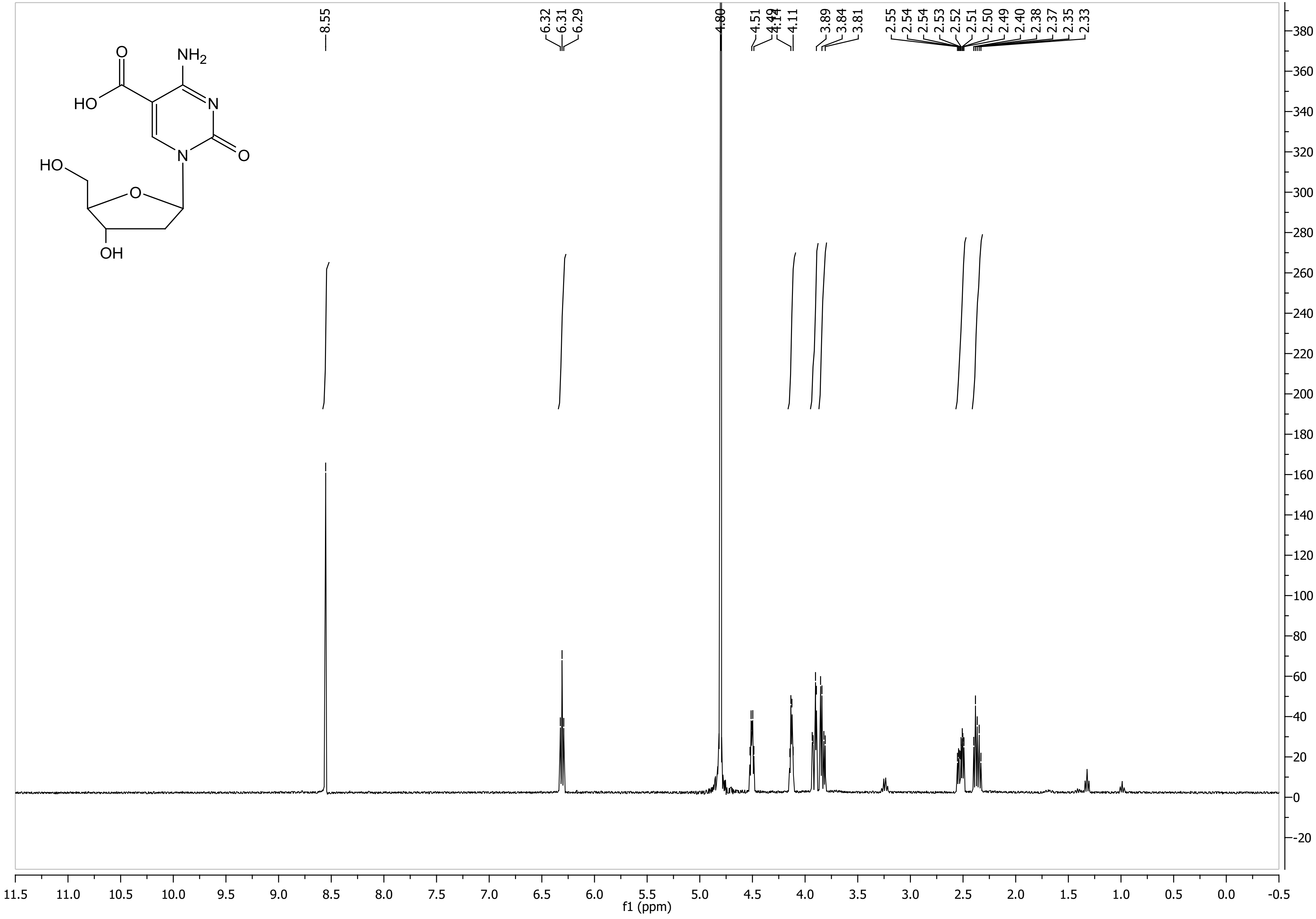
8.55
6.32
6.31
6.29
4.80
4.51
4.11
3.89
3.84
3.81
2.55
2.54
2.54
2.53
2.52
2.51
2.50
2.49
2.40
2.38
2.37
2.35
2.33
f1 (ppm)
NH2
HO
O
N
OH

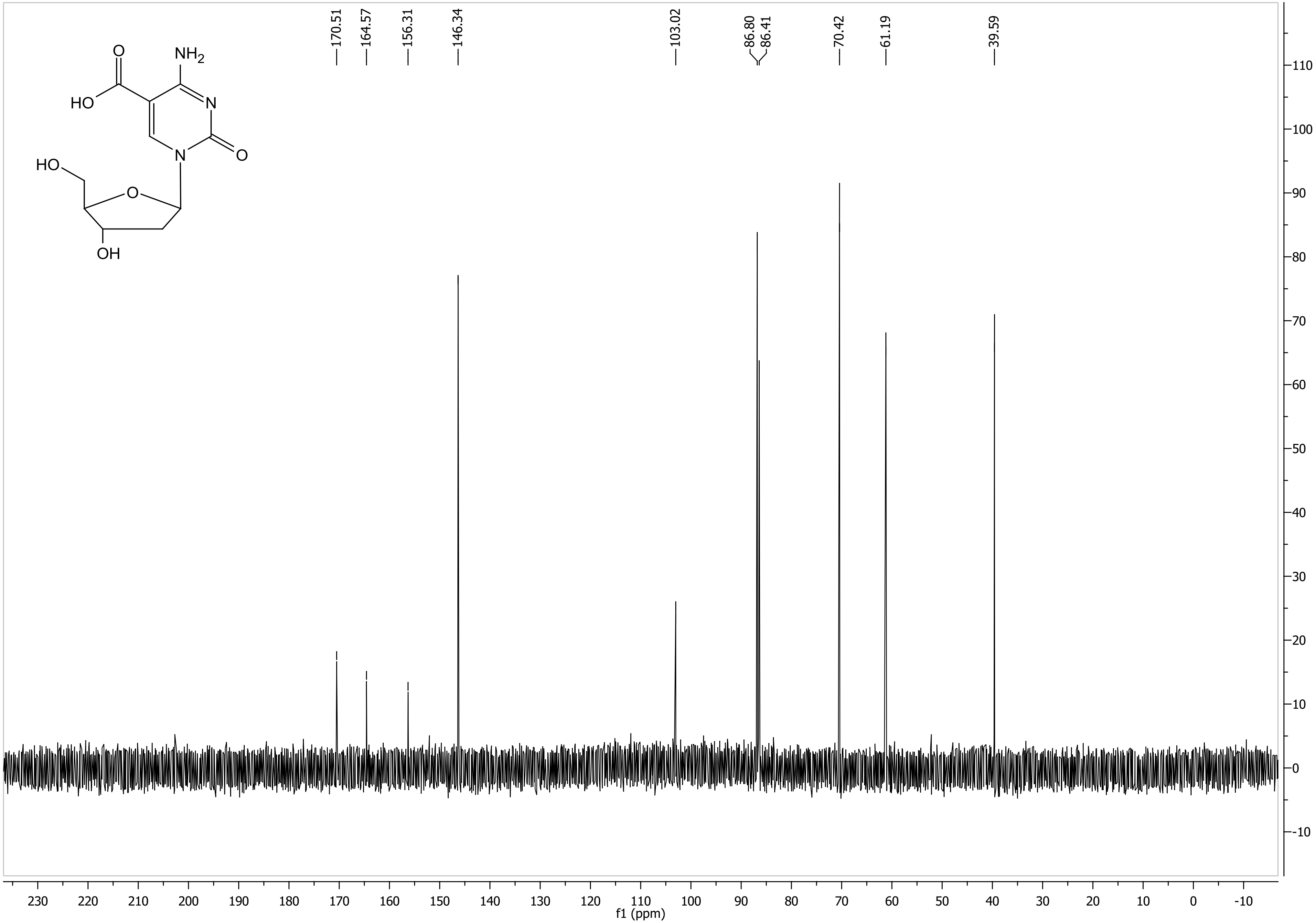
170.51
164.57
156.31
146.34
103.02
86.80
86.41
70.42
61.19
39.59
NH2
HO
O
N
HO
OH
f1 (ppm)

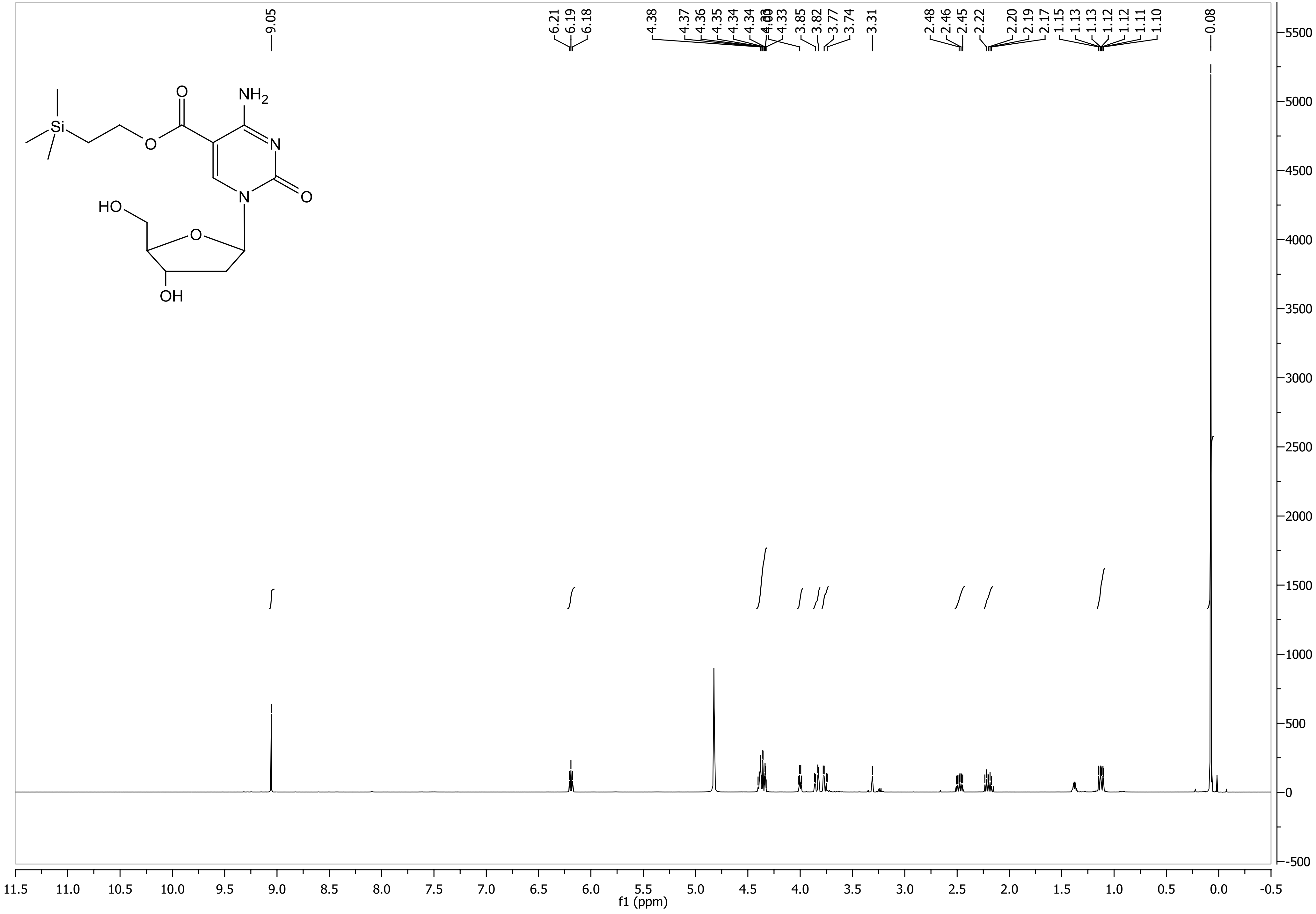
9.05
6.21
6.19
6.18
4.38
4.37
4.36
4.35
4.34
4.34
4.33
3.85
3.82
3.77
3.74
3.31
2.48
2.46
2.45
2.22
2.20
2.19
2.17
1.15
1.13
1.13
1.12
1.12
1.11
1.10
0.08
NH2
O
N
Si
HO
OH
f1 (ppm)

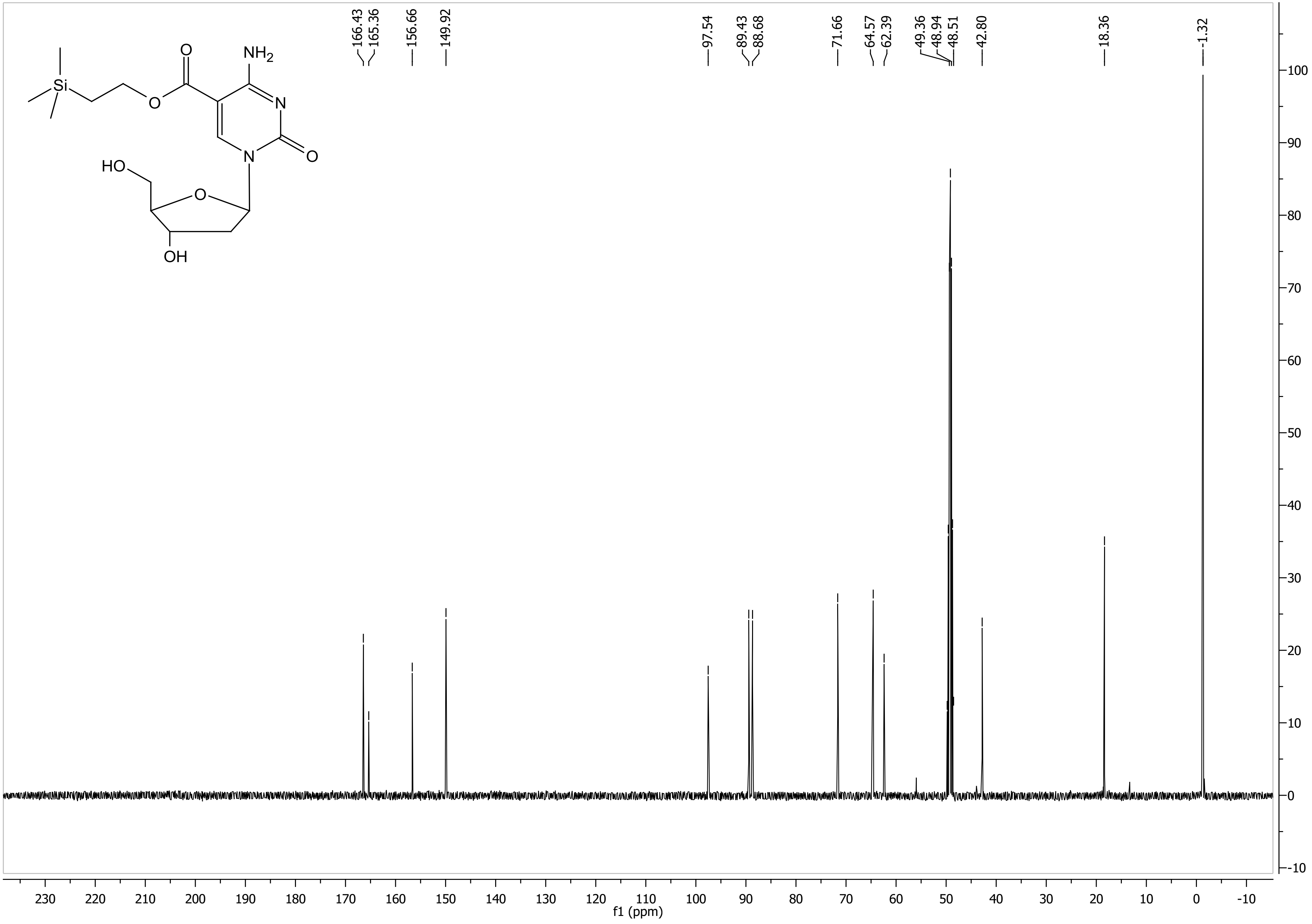

NH2
O
N
O
Si
O
N
HO
O
OH
166.43
165.36
156.66
149.92
97.54
89.43
88.68
71.66
64.57
62.39
49.36
48.94
48.51
42.80
18.36
-1.32
f1 (ppm)

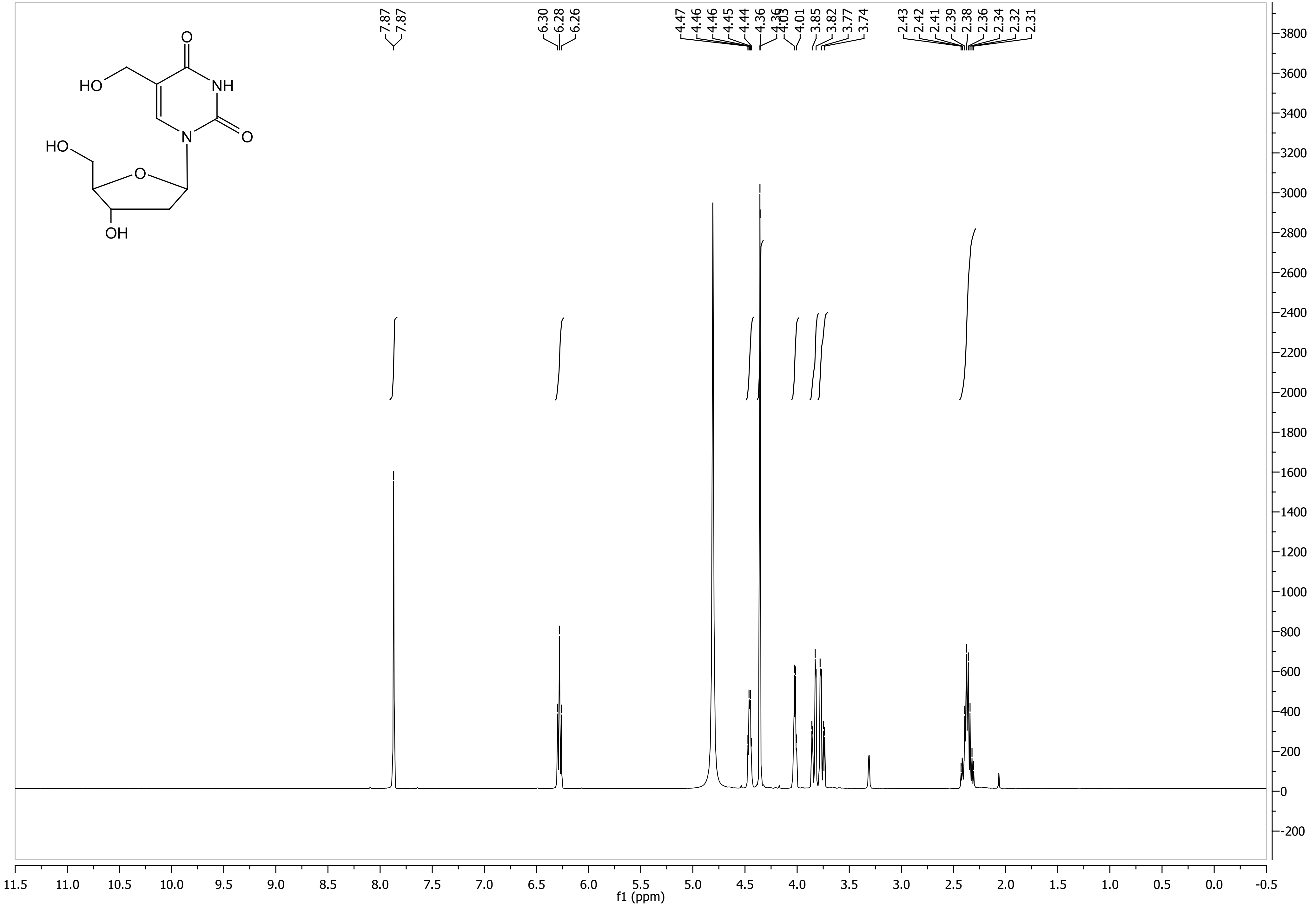

7.87
7.87
6.30
6.28
6.26
4.47
4.46
4.46
4.45
4.44
4.36
4.36
4.03
4.01
3.85
3.82
3.77
3.74
2.43
2.42
2.41
2.39
2.38
2.36
2.34
2.32
2.31
HO
NH
O
N
HO
O
OH
f1 (ppm)

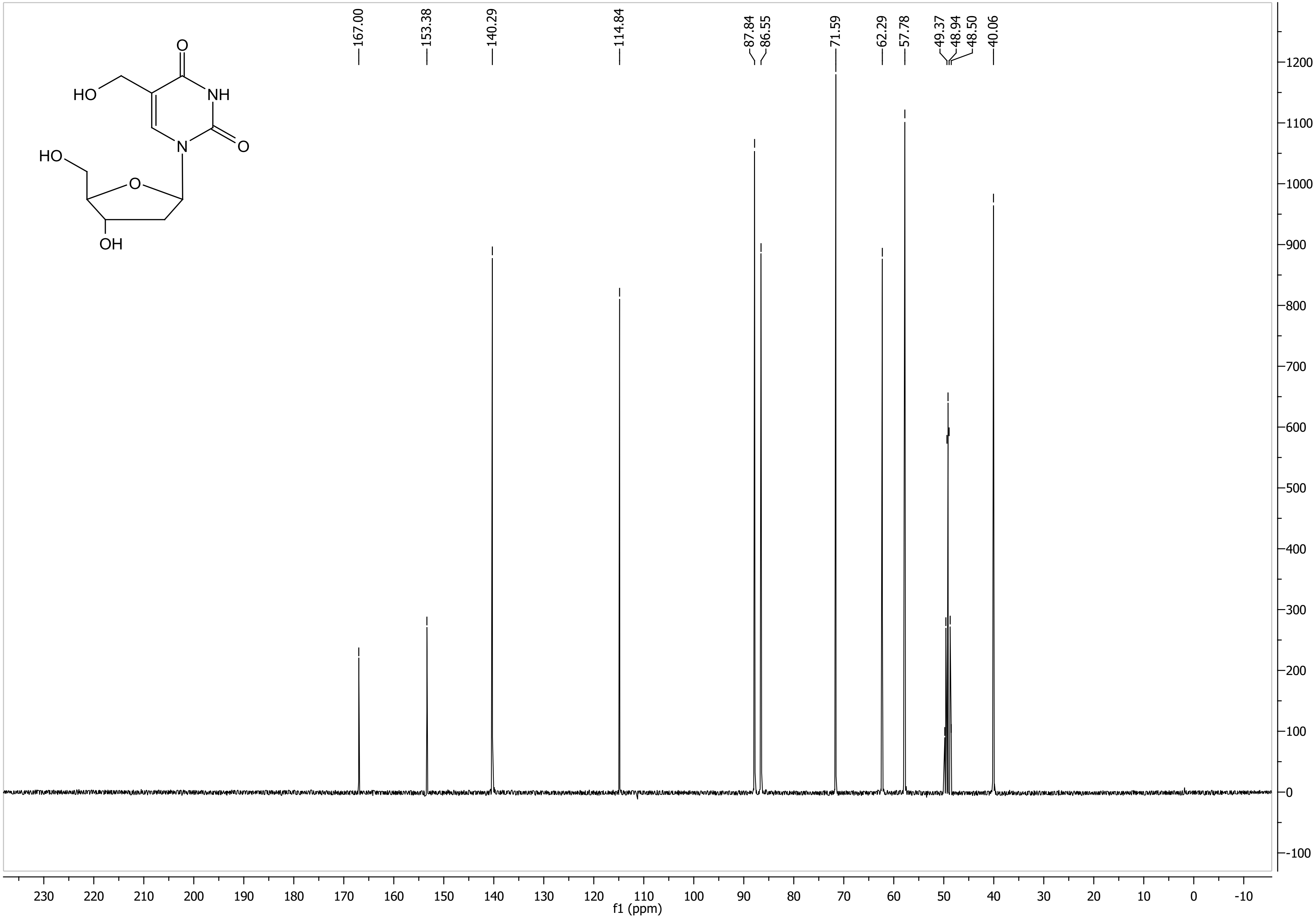

167.00
153.38
140.29
114.84
87.84
86.55
71.59
62.29
57.78
49.37
48.94
48.50
40.06
HO
NH
O
N
OH
f1 (ppm)
230 220 210 200 190 180 170 160 150 140 130 120 110 100 90 80 70 60 50 40 30 20 10 0 -10
1200 1100 1000 900 800 700 600 500 400 300 200 100 0 -100
